# Delineating the “who-where-how” of persistent HIV epidemics: a 23-year longitudinal genetic network, phylodynamic, and spatial analysis for precise intervention in rural China

**DOI:** 10.1080/22221751.2026.2671470

**Published:** 2026-05-13

**Authors:** Yi Chen, Xiaoshan Xu, Jianjun Li, Shujia Liang, Jinghua Huang, Kailing Tang, Hui Xing, Lingjie Liao, Yi Feng, Jingjun Huang, Wanling Chen, Yuhua Ruan, Ge Zhong

**Affiliations:** a The Guangxi Academy of Medical Sciences, The People’s Hospital of Guangxi Zhuang Autonomous Region, Nanning, People’s Republic of China; b State Key Laboratory of Infectious Disease Prevention and Control (SKLID), Collaborative Innovation Center for Diagnosis and Treatment of Infectious Diseases, National Center for AIDS/STD Control and Prevention (NCAIDS), Chinese Center for Disease Control and Prevention (China CDC), Beijing, People’s Republic of China; c Guangxi Center for Disease Control and Prevention, Nanning, People’s Republic of China; d School of Public Health and Management, Guangxi University of Chinese Medicine, Nanning, People’s Republic of China

**Keywords:** Longitudinal HIV genetic network, phylogeographic analysis, spatial epidemiology, “Treat All” policy, precision public health

## Abstract

Despite “Treat All” strategies, HIV outbreaks persist among the elderly in rural Southwest China. To identify the core drivers (“Who”), geographic foci (“Where”), and socio-behavioural mechanism (“How”) of sustained transmission in the post-“Treat All” era, we triangulated longitudinal genetic networks, phylodynamic and spatial analysis, and behavioural surveys using multi-source 1999–2021 data (*N* = 5094). Although antiretroviral therapy reduced community-level transmission risk by 21.0%, 107 molecular super-spreaders, predominantly males aged 50–69, were identified across four phases. These individuals exhibited poor treatment retention, with dropout rates reaching 28.2% and 38.5% having unsuppressed or missing viral loads by Phase 4. Notably, 94.4% of super-spreaders resided within 2 km of transportation arteries, exceeding 77.9% population baseline. Townships within this buffer exhibited significantly higher cumulative HIV cases independently of population metrics (adjusted Incidence Rate Ratio = 1.43, 95% CI: 1.14–1.78). Characterized as infrastructure-anchored “risk hubs,” these areas facilitate sustained viral dispersal through a “double-proximity” mechanism. Older men frequent nearby periodic markets along transit corridors for commercial sex, while female sex workers exhibit linear mobility across townships, effectively aligning their service cycles with the temporal rhythm of the periodic markets. Our methodology facilitates targeted node-level testing and interventions, bridging clinical treatment and public health prevention to mitigate aging-driven epidemics in resource-limited regions.

## Background

The global scale-up of “Treat All” antiretroviral therapy (ART) policy represents a monumental achievement in the fight against HIV/AIDS, aiming to reduce mortality and interrupt transmission at the population level [1–3]. Yet, the persistence of localized HIV outbreaks poses a critical challenge to achieve universal epidemic control [4,5]. This implementation gap suggests that the public health impact of “Treat All” might be undermined not by drug efficacy, but by the existence of HIV super-spreaders and substantial silent reservoirs. These reservoirs, comprised of undiagnosed HIV-positive cases and high-risk individuals, remain beyond the reach of conventional screening [5].

This challenge was acutely evident in rural southwest China. Despite the nationwide rollout of “Treat All” in 2016 and significant progress under the Guangxi AIDS Conquering Project (GACP, 2010–2020), featuring expanded ART coverage (scaling from 56.1% in 2016 to 86.7% by 2021), intensified education, and voluntary testing, which collectively decelerated epidemic growth [6,7], persistent high-risk behaviours, particularly unprotected sex, continued to fuel transmission, especially among the older population [8]. The number of newly diagnosed HIV/AIDS cases in Guangxi remained at approximately 10,000 annually over the past five years, accounting for roughly 10% of the national total; of the newly diagnosed, heterosexuals continually accounted for over 90.0% [9], and the proportion of newly diagnosed aged ≥50 years increased sharply from 37.3% in 2010 to 67.2% in 2024, significantly exceeding the national average during the same period. Given that a small fraction of cases drives the majority of infections, precision-targeted interventions are essential to maximize public health returns [10].

However, precisely defining the “Who” and “Where” of these super-spreaders and “silent reservoirs,” as well as “How” they make the epidemic persistent, remains a formidable challenge. Longitudinal viral phylogenetic data, which captures the underlying transmission network, offers a novel lens for such an audit [11,12]. When integrated with spatial analysis and behavioural surveys, this approach moves beyond a simple census of diagnosed individuals to provide mechanistic insights into “Where” transmission is most active, “Who” is driving it, and “How” these dynamics sustain the epidemic. Yet, to date, most studies have relied on fragmented methodologies. For instance, spatial clustering based solely on reported cases [13,14] lacks molecular evidence to verify cross-regional linkages. Similarly, cross-sectional genetic “snapshots” [15–18] offer limited insight into the epidemic’s long-term temporal evolution. Even recent advancements combining genetic networks with spatial analysis [19] remain largely descriptive, providing a static mapping of genetic linkages without capturing the longitudinal momentum of the epidemic’s expansion. Furthermore, although macro-level phylogeographic studies [20] had traced CRF07_BC lineages across broad provincial boundaries, such findings remain too coarse-grained to guide the localized, high-precision interventions necessary at the community level.

To bridge these gaps, we conducted a 23-year (1999–2021) longitudinal study in rural Southwest China, implementing a multidimensional framework that integrated Generalized Estimating Equations (GEE) modelling, Bayesian phylogeographic reconstruction, and longitudinal genetic networks with spatiotemporal clustering and supplementary exploratory behavioural surveys. Moving beyond static surveillance, this integrated approach enables us to evaluate the population-level impact of the “Treat All” policy while unmasking the “Who-Where-How” of the transmission engines and “silent reservoirs” that disproportionately sustain viral dispersal. By synthesizing these diverse data layers, we aimed to provide a robust evidence base for high-precision, context-specific interventions capable of neutralizing the hidden drivers and reversing the aging-associated trajectory of the HIV epidemic in rural Southwest China.

## Materials and methods

### Study setting and design

Guangxi is a high-prevalence region, with an HIV prevalence (0.25%) nearly triple the national average (0.09%) [21]. As one of China’s top three provinces for new HIV/AIDS diagnoses, it epitomizes the unique epidemic trajectories observed in rural Southwest China that the HIV epidemic transitioned from 1990s injecting drug user (IDU)-driven clusters to current elderly-dominated heterosexual transmission [22]. Within this context, Qinzhou, a strategic coastal port city and transportation hub, was selected as the study site, as it ranks among the top three prefectures for HIV burden in the province. It serves as a representative model for regional HIV-1 dynamics [7,22]. This study retrospectively analysed cases diagnosed from 1999 to 2021. Ethical approval was obtained from the Institutional Review Board (IRB) of the National Center for AIDS/STD Control and Prevention (NCAIDS), Chinese Center for Disease Control and Prevention (China CDC) (No. X221109713). The requirement for informed consent was waived by the ethics committee due to the study’s retrospective nature and the use of de-identified data.

### Study population and sample selection

The study population comprised all newly diagnosed HIV/AIDS cases in Qinzhou between 1999 and 2021. Residual serum specimens of these cases were obtained from routine HIV confirmatory tests (for ART-naïve cases) or CD4 count assessment (for ART-experienced cases). HIV-1 Ribonucleic Acid (RNA) was sequenced from pre-ART plasma samples cryopreserved at −80°C. For patients undergoing ART, pro-viral Deoxy ribonucleic Acid (DNA) was extracted from whole blood to ensure sequence recovery despite viral suppression. From this initial cohort, cases were excluded based on the following criteria: (i) age < 18 years; (ii) insufficient specimen volume; (iii) poor sequence quality (e.g. amplification failure, ≥ 5% mixed bases, pol sequences < 1000 bp, or RT length < 238 codons); and (iv) incomplete epidemiological records. All nucleic acid extraction, amplification, and sequencing were performed at the Guangxi CDC HIV/AIDS Confirmatory Laboratory following established protocols [7,23,24].

### Epidemiological data collection

The corresponding de-identified demographic and epidemiological data for the included cases were extracted from the Guangxi HIV/AIDS Comprehensive Response Information Management System of China’s National System. The data encompassed age, gender, ethnicity, education, marital status, occupation, residence, HIV transmission route (risk), date of HIV diagnosis, baseline CD4 level (defined as the CD4 counts at HIV diagnosis), HIV viral load, and ART status (categorized as ART-naïve, active ART, or ART dropout). Dropout was assessed longitudinally and defined as treatment interruption or loss to follow-up for > 90 days since the last scheduled visit [25] . Patients who transferred to other ART authorities with confirmed referrals were continued as “active ART,” while those with unverifiable status post-migration were classified as dropouts.

### Epidemiological phasing

The study period was stratified into four distinct epidemiological phases: (1) Phase I (1999–2009): Pre-GACP phase, (2) Phase II (2010–2015): GACP intensive intervention phase, (3) Phase III (2016–2018): “Treat All” transition phase, and (4) Phase IV (2019–2021): “Treat All” full coverage phase. The rationale of the stratification is provided in Supplementary File 2 “1. Stratification of four epidemiological phases.”

### HIV genetic network reconstruction and topological importance quantified

HIV genetic networks were reconstructed using all available sequences (*N* = 5094) spanning four epidemiological phases (1999–2021). Pairwise genetic distances were calculated using HyPhy (version 2.2.4) under the Tamura-Nei 93 (TN93) model. Putative transmission links were identified using a stringent threshold of 0.005 substitutions/site to capture recent (2–3 years) transmission events [26]. For each phase, township-level topological importance was quantified by calculating global parameters (nodes, edges, density, and number/size of clusters) and centrality indices (mean degree, betweenness, and eigenvector centrality). Individuals whose degree centrality exceeded the mean by two standard deviations (μ + 2σ) within each respective phase were defined as molecular super-spreaders. This stringent statistical threshold ensures the identification of extreme outliers with the most significant impact on network expansion while accounting for temporal variations in network size and connection density across the four epidemiological phases. To protect participant privacy, specific geographic identifiers (e.g. township and street names) were redacted from the manuscript. However, these granular data were retained for internal geospatial analysis to identify high-risk clusters and inform local interventions. This approach balances epidemiological utility with strict confidentiality.

### BEAST-based phylogeographic analysis

HIV-1 subtype identification was based on MAGE (v10.0). Bayesian phylogeographic analysis was performed based on RNA sequences of five major HIV subtypes/clusters using BEAST 1.8.4. The specific subtype distribution for these RNA sequences was illustrated in Supplementary file 1 Table S1. As detailed in Supplementary File 2 “ 2. Phylodynamic and phylogeographic analysis,” we processed sequences and configured BEAST models to investigate the epidemic’s trajectory. Specifically, we employed a phylogenetic time-slicing approach to quantify temporal shifts in the demographic composition of each lineage across four epidemiological phases, visualized using smoothed stacked area charts [27]. In parallel, Bayesian stochastic search variable selection (BSSVS) was applied to infer viral migration dynamics between subgroups [28].

### Geospatial and genetic network integration analysis

Geospatial distribution of transmission hubs was analysed by aggregating genetic network features at the township level (georeferenced by geometric centroids), visualized using ArcGIS 10.8. Intra-township links and inter-township connections were visualized to represent localized and regional connectivity, respectively. Specifically, mean degree centrality and Mean Molecular Cluster Growth (MMCG) were used to quantify connectivity and expansion intensity. MMCG’s definition and calculation was described in Supplementary file 2 “ 3. Mean Molecular Cluster Growth calculation.” These metrics were further integrated with Geographic Information System (GIS) data, utilizing road proximity and spatial autocorrelation, to evaluate the impact of transportation infrastructure on viral dissemination. Negative Binomial regression analysis was further performed to isolate the impact of road proximity from the effects of general population aggregation. Detailed methods for spatial autocorrelation and road proximity analysis were provided in Supplementary file 2 “ 4. Spatial autocorrelation analysis” and “ 5. Road proximity analysis.”

### A supplementary exploratory behavioural survey

To explore the socio-behavioural mechanism of the observed molecular and spatiotemporal patterns, we conducted a supplementary exploratory behavioural survey targeted at local men (N = 84) across three townships along the major transportation corridors in June-July 2025. A hybrid sampling strategy was employed. Initially, 22 diagnosed HIV cases were recruited as seeds for potential chain-referral; subsequently, to ensure broader community representation, we integrated venue-based sampling at traditional informal social gathering points (e.g. village entrances and community parks) frequented by older adults. Socio-demographic profiles, the frequency and spatial context of non-marital sexual encounters (including both commercial and casual sex), and condom use behaviours were collected from all participants. Additionally, data regarding the geographic location and mobility status of the encountered female sex workers (FSWs) were recorded. To ensure maximum participant protection and minimize social stigmatization, verbal informed consent was obtained in lieu of written documentation, and all data were de-identified and restricted to authorized public health personnel to maintain strict confidentiality. The interviews were approved by the IRB of the NCAIDS, China CDC (IRB No. X221109713).

### Statistical analysis

Analyses were performed in R (version 4.3.2) using sf for spatial processing, spdep for autocorrelation metrics and ggplot2 for visualization. Chi-square tests were used to compare categorical variables. A GEE model was used to assess the association between ART status and onward HIV transmission among all samples (N = 5094), details in Supplementary file 2 “6. Generalized estimating equations analysis.” A two-tailed *P* < 0.05 was considered significant.

## Results

### Epidemiological landscape and cohort characteristics (1999–2021)

From 1999 to 2021, a total of 7381 HIV/AIDS cases were newly diagnosed in the study area. 69.0% (*n* = 5094) met the inclusion criteria with high-quality sequence and complete epidemiological records. This sequencing cohort comprised 4814 plasma RNA sequences and 280 pro-viral DNA sequences (Supplementary file 1 Figure S1). Demographic comparison confirmed that the 4814-case RNA sub-cohort was statistically representative of the overall diagnosed population (all *P* > 0.05; Supplementary file 1 Table S4), thus validating its use for subsequent Bayesian phylodynamic (BEAST) analysis. As shown in Table 1, the overall study population was primarily composed of males (74.6%), cases aged over 50 years old (52.9%), Han ethnicity (89.6%), and farmers (75.0%), with heterosexual transmission (89.9%) being the predominant route. Over the 23-year span, the cohort exhibited a robust aging trend, with the proportion of cases aged 50–69 surging from 11.8% to 51.0% (*P _trend_* < 0.001). The proportion of divorced/widowed cases rose from 11.5% to 25.4% (*P _trend_* < 0.001). Further, a near-complete transition from injecting drug use to heterosexual transmission was observed (*P _trend_* < 0.001). At the molecular level, these changes mirrored a transition towards a more diversified viral reservoir. While CRF01_AE and CRF08_BC remained the dominant strains in absolute numbers, their proportional contributions significantly contracted (*P _trend_* < 0.001). Conversely, CRF07_BC experienced rapid expansion, increasing from 4.5% to 14.9% (*P _trend_* < 0.001). Although CRF55_01B also showed a statistically significant upward trend (*P _trend_* < 0.001), its overall prevalence remained below 2.0% throughout the study period (Details see Table 1 and Supplementary file 1 Figure S2).

**Table 1.** Basic social-demographic characteristics of all participants (*N* = 5094).

	Total	1999–2009	2010–2015	2016	2017	2018	2019	2020	2021	*X*^2^ (Overall *P)*	*P _trend_*
Total	5094 (100.0)	288 (100.0)	1139 (100.0)	698 (100.0)	597 (100.0)	602 (100.0)	674 (100.0)	525 (100.0)	571 (100.0)		
Age (Years)										401.16 (< 0.001)	
18–24	184 (3.6)	2 (0.7)	52 (4.6)	23 (3.3)	31 (5.2)	24 (4.0)	20 (3.0)	14 (2.7)	18 (3.2)		0.816
25–34	725 (14.2)	95 (33.0)	230 (20.2)	105 (15.0)	75 (12.6)	72 (12.0)	68 (10.1)	43 (8.2)	37 (6.5)		**< 0.001**
35–49	1491 (29.3)	151 (52.4)	366 (32.1)	206 (29.5)	176 (29.5)	147 (24.4)	179 (26.6)	131 (25.0)	135 (23.6)		**< 0.001**
50–69	2192 (43.0)	34 (11.8)	386 (33.9)	303 (43.4)	262 (43.9)	300 (49.8)	340 (50.4)	276 (52.6)	291 (51.0)		**< 0.001**
≥70	502 (9.9)	6 (2.1)	105 (9.2)	61 (8.7)	53 (8.9)	59 (9.8)	67 (9.9)	61 (11.6)	90 (15.8)		**< 0.001**
Gender										24.69 (< 0.001)	
Female	1296 (25.4)	47 (16.3)	269 (23.6)	198 (28.4)	154 (25.8)	153 (25.4)	200 (29.7)	127 (24.2)	148 (25.9)		**< 0.001**
Male	3798 (74.6)	241 (83.7)	870 (76.4)	500 (71.6)	443 (74.2)	449 (74.6)	474 (70.3)	398 (75.8)	423 (74.1)		**< 0.001**
Ethnicity										32.14 (< 0.001)	
Han	4564 (89.6)	269 (93.4)	1026 (90.1)	626 (89.7)	537 (89.9)	533 (88.5)	597 (88.6)	493 (93.9)	483 (84.6)		0.009
Other	530 (10.4)	19 (6.6)	113 (9.9)	72 (10.3)	60 (10.1)	69 (11.5)	77 (11.4)	32 (6.1)	88 (15.4)		0.009
Education										54.31 (< 0.001)	
Illiteracy	236 (4.6)	8 (2.8)	43 (3.8)	36 (5.2)	25 (4.2)	26 (4.3)	42 (6.2)	21 (4.0)	35 (6.1)		0.042
Elementary school	2602 (51.1)	115 (39.9)	574 (50.4)	351 (50.3)	311 (52.1)	300 (49.8)	350 (51.9)	299 (57.0)	302 (52.9)		**< 0.001**
Junior school	1830 (35.9)	147 (51.0)	428 (37.6)	248 (35.5)	206 (34.5)	226 (37.5)	228 (33.8)	168 (32.0)	179 (31.3)		**< 0.001**
High school and above	426 (8.4)	18 (6.3)	94 (8.3)	63 (9.0)	55 (9.2)	50 (8.3)	54 (8.0)	37 (7.0)	55 (9.6)		0.161
Marital status										138.83 (< 0.001)	
Single	1178 (23.1)	130 (45.1)	314 (27.6)	125 (17.9)	123 (20.6)	120 (19.9)	133 (19.7)	108 (20.6)	125 (21.9)		**< 0.001**
Married	2903 (57.0)	125 (43.4)	620 (54.4)	448 (64.2)	369 (61.8)	352 (58.5)	393 (58.3)	295 (56.2)	301 (52.7)		**< 0.001**
Divorced/Widowed	1013 (19.9)	33 (11.5)	205 (18.0)	125 (17.9)	105 (17.6)	130 (21.6)	148 (22.0)	122 (23.2)	145 (25.4)		**< 0.001**
Occupation										263.20 (< 0.001)	
Farmer	3822 (75.0)	119 (41.3)	827 (72.6)	554 (79.4)	445 (74.5)	476 (79.1)	532 (78.9)	418 (79.6)	451 (79.0)		**< 0.001**
House worker	653 (12.8)	76 (26.4)	158 (13.9)	67 (9.6)	71 (11.9)	68 (11.3)	76 (11.3)	72 (13.7)	65 (11.4)		**< 0.001**
Retired	91 (1.8)	2 (0.7)	26 (2.3)	17 (2.4)	15 (2.5)	10 (1.7)	12 (1.8)	4 (0.8)	5 (0.9)		0.887
Other	528 (10.4)	91 (31.6)	128 (11.2)	60 (8.6)	66 (11.1)	48 (8.0)	54 (8.0)	31 (5.9)	50 (8.8)		**< 0.001**
Risk										1288.24 (< 0.001)	
Injecting drug	419 (8.2)	181 (62.8)	123 (10.8)	26 (3.7)	18 (3.0)	29 (4.8)	16 (2.4)	14 (2.7)	12 (2.1)		**< 0.001**
Heterosexual	4581 (89.9)	107 (37.2)	1002 (88)	658 (94.3)	560 (93.8)	562 (93.4)	643 (95.4)	504 (96.0)	545 (95.4)		**< 0.001**
Homosexual	94 (1.8)	0 (0.0)	14 (1.2)	14 (2.0)	19 (3.2)	11 (1.8)	15 (2.2)	7 (1.3)	14 (2.5)		**< 0.001**
Residence										107.02 (< 0.001)	
Qinnan	1056 (20.7)	39 (13.5)	261 (22.9)	145 (20.8)	128 (21.4)	123 (20.4)	156 (23.1)	79 (15.0)	125 (1.9)		> 0.050
Qinbei	1083 (21.3)	61 (21.2)	227 (19.9)	163 (23.4)	143 (24.0)	123 (20.4)	152 (22.6)	81 (15.4)	133 (23.3)		> 0.050
Lingshan	2283 (44.8)	168 (58.3)	512 (45.0)	273 (39.1)	234 (39.2)	284 (47.2)	279 (41.4)	300 (57.1)	233 (40.8)		0.020
Pubei	635 (12.5)	20 (6.9)	126 (11.1)	111 (15.9)	87 (14.6)	71 (11.8)	82 (12.2)	64 (12.2)	74 (13.0)		**< 0.001**
Kaifaqu	37 (0.7)	0 (0.0)	13 (1.1)	6 (0.9)	5 (0.8)	1 (0.2)	5 (0.7)	1 (0.2)	6 (1.1)		> 0.050
Subtype/Cluster										450.48 (< 0.001)	
CRF01_AE Cluster 1	549 (10.8)	23 (8.0)	154 (13.5)	81 (11.6)	73 (12.2)	53 (8.8)	66 (9.8)	46 (8.8)	53 (9.3)		**0.001**
CRF01_AE Cluster 2	1858 (36.5)	70 (24.3)	511 (44.9)	296 (42.4)	239 (40.0)	227 (37.7)	241 (35.8)	147 (28.0)	127 (22.2)		**< 0.001**
CRF07_BC	516 (10.1)	13 (4.5)	71 (6.2)	57 (8.2)	73 (12.2)	52 (8.6)	86 (12.8)	79 (15.0)	85 (14.9)		**< 0.001**
CRF08_BC	1619 (31.8)	148 (51.4)	305 (26.8)	187 (26.8)	149 (25.0)	197 (32.7)	217 (32.2)	192 (36.6)	224 (39.2)		**< 0.001**
CRF55_01B	55 (1.1)	0 (0.0)	4 (0.4)	6 (0.9)	10 (1.7)	16 (2.7)	4 (0.6)	4 (0.8)	11 (1.9)		**< 0.001**
Others	497 (9.8)	34 (11.8)	94 (8.3)	71 (10.2)	53 (8.9)	57 (9.5)	60 (8.9)	57 (10.9)	71 (12.4)		0.040

Notes: *X*^2^ (Overall *P*) represents the results of Pearson’s chi-square test for overall distribution across study periods. *P_trend_* denotes the significance of linear trends for specific categories, calculated via the Cochran-Armitage trend test.

### Population-level protection of the “Treat All” policy

The implementation of the nationwide “Treat All” policy in 2016 provided valuable real-world evidence to evaluate the impact of immediate ART. Although its effective execution in rural areas exhibited a temporal lag, the population-level efficacy remains profound. GEE analysis based on a 5094-sequence dataset with 19,661 observations shows that active ART was associated with a significant 21% reduction in HIV secondary transmission [Adjusted odds ratio (AOR) = 0.79, 95% CI: 0.73–0.87], while ART dropout showed no significant protective effect compared to being ART-naïve (see Table 2). However, GEE identified several factors independently associated with an increased risk of secondary transmission, most notably older age, which exhibited a graded increase in risk [35–49 years, AOR = 1.60 (95%CI: 1.20–2.14); 50–69 years, AOR = 3.59 (95%CI: 2.68–4.81); ≥70 years, AOR = 4.22 (95% CI: 3.09–5.77), all vs. < 25 years], male gender (AOR = 1.42, 95% CI: 1.29–1.56), infected with CRF07_BC (AOR = 1.94, 95% CI: 1.71–2.21) or CRF08_BC subtypes (AOR = 1.75, 95% CI: 1.59–1.92), residence in Qinbei (AOR = 1.17, 95% CI: 1.05–1.31), and higher baseline CD4 level [350–499 vs. < 350: AOR = 1.23 (95%CI: 1.10–1.37); ≥500 vs. <350: AOR = 1.26 (95%CI: 1.10–1.45)], see Table 2.

**Table 2.** Factors associated with HIV molecular linkage between baseline cases (1999–2016) and newly diagnosed cases (2017–2021): a generalized estimating equation analysis (*N* = 5094; Observations = 19,661).

Variable	Observations	≥ 1 (%)	1 (%)	≥ 2 (%)	OR (95%CI)	*P* value	AOR (95%CI)	*P* value
Total	19,661	3463 (17.6)	1567 (8.0)	1896 (9.6)				
ART status								
ART-naive	6292	1297 (20.6)	577 (9.2)	720 (11.4)	Ref.		Ref.	
Active ART	12,377	1984 (16.0)	921 (7.4)	1063 (8.6)	0.74(0.68–0.80)	< 0.001	0.79(0.73–0.87)	< 0.001
ART dropout	992	182 (18.3)	69 (7.0)	113 (11.4)	0.87(0.73–1.03)	0.132	0.90(0.75–1.08)	0.274
Age (years)								
<25	742	63 (8.5)	39 (5.3)	24 (3.2)	Ref.		Ref.	
25–34	3140	220 (7.0)	134 (4.3)	86 (2.7)	0.81 (0.61–1.09)	0.163	0.89(0.66–1.21)	0.465
35–49	6017	694 (11.5)	340 (5.7)	354 (5.9)	1.41 (1.07–1.84)	0.014	1.60(1.20–2.14)	0.001
50–69	7988	1930 (24.2)	822 (10.3)	1108 (13.9)	3.43 (2.64–4.47)	< 0.001	3.59(2.68–4.81)	< 0.001
≥70	1774	556 (31.3)	232 (13.1)	324 (18.3)	4.92 (3.73–6.49)	< 0.001	4.22(3.09–5.77)	< 0.001
Gender								
Female	4954	730 (14.7)	356 (7.2)	374 (7.5)	Ref.		Ref.	
Male	14,707	2733 (18.6)	1211 (8.2)	1522 (10.3)	1.32 (1.21–1.44)	< 0.001	1.42(1.29–1.56)	< 0.001
Education								
Illiteracy	867	229 (26.4)	89 (10.3)	140 (16.1)	Ref.		Ref.	
Elementary school	7248	1099 (15.2)	508 (7.0)	591 (8.2)	0.66 (0.56–0.77)	< 0.001	0.85(0.72–1.01)	0.072
Junior school	9905	1897 (19.2)	859 (8.7)	1038 (10.5)	0.50 (0.42–0.59)	< 0.001	0.81(0.68–0.98)	0.027
High school and above	1641	238 (14.5)	111 (6.8)	127 (7.7)	0.47 (0.39–0.58)	< 0.001	0.67(0.53–0.84)	0.001
Marital status								
Single	4680	578 (12.4)	302 (6.5)	276 (5.9)	Ref.		Ref.	
Married	11,288	2145 (19.0)	951 (8.4)	1194 (10.6)	1.66 (1.51–1.84)	< 0.001	0.97(0.86–1.09)	0.614
Divorced/Widowed	3693	740 (20.0)	314 (8.5)	426 (11.5)	1.78 (1.58–2.00)	< 0.001	0.90(0.78–1.03)	0.131
Ethnicity								
Han	17,682	3028 (17.1)	1397 (7.9)	1631 (9.2)	Ref.		Ref.	
Other	1979	435 (22.0)	170 (8.6)	265 (13.4)	1.36(1.22–1.53)	< 0.001	1.09(0.96–1.25)	0.179
Occupation								
Farmer	14,512	2671 (18.4)	1212 (8.4)	1459 (10.1)	Ref.		Ref.	
House worker	2569	419 (16.3)	202 (7.9)	217 (8.4)	0.86 (0.77–0.97)	< 0.001	1.10(0.97–1.25)	0.141
Retired	389	118 (30.3)	43 (11.1)	75 (19.3)	1.93 (1.55–2.41)	< 0.001	1.18(0.93–1.51)	0.172
Other	2191	255 (11.6)	110 (5)	145 (6.6)	0.58 (0.51–0.67)	< 0.001	0.83(0.71–0.98)	0.023
Risk								
Injection drug use	1944	122 (6.3)	78 (4)	44 (2.3)	Ref.		Ref.	
Heterosexual	17,365	3307 (19.0)	1469 (8.5)	1838 (10.6)	3.51 (2.91–4.24)	< 0.001	2.64(2.13–3.27)	< 0.001
Homosexual	352	34 (9.7)	20 (5.7)	14 (4.0)	1.60 (1.07–2.38)	0.021	2.25(1.45–3.48)	< 0.001
Location								
Qinnan	4108	864 (21.0)	370 (9.0)	494 (12.0)	Ref.		Ref.	
Qinbei	4213	969 (23.0)	368 (8.7)	601 (14.3)	1.12 (1.01–1.24)	0.030	1.17(1.05–1.31)	0.006
Lingshan	8741	1243 (14.2)	651 (7.4)	592 (6.8)	0.62 (0.57–0.69)	< 0.001	0.67(0.60–0.75)	< 0.001
Pubei	2452	353 (14.4)	162 (6.6)	191 (7.8)	0.63 (0.55–0.72)	< 0.001	0.65(0.57–0.75)	< 0.001
Kaifaqu	147	34 (23.1)	16 (10.9)	18 (12.2)	1.13 (0.76–1.67)	0.540	1.22(0.80–1.85)	0.355
Subtype								
CRF01_AE	9942	1575 (15.8)	790 (7.9)	785 (7.9)			Ref.	
CRF07_BC	1779	439 (24.7)	152 (8.5)	287 (16.1)	1.74 (1.54–1.96)	< 0.001	1.94(1.71–2.21)	< 0.001
CRF08_BC	5992	1142 (19.1)	512 (8.5)	630 (10.5)	1.25 (1.15–1.36)	< 0.001	1.75(1.59–1.92)	< 0.001
Other	1948	307 (15.8)	113 (5.8)	194 (10.0)	0.99 (0.87–1.14)	0.928	1.42(1.23–1.63)	< 0.001
Baseline CD4 level								
<350	14,632	12,088 (61.5)	1170 (6.0)	1374 (7.0)				
350–499	2931	2378 (12.1)	232 (1.2)	321 (1.6)	1.10 (1.00–1.22)	0.055	1.23(1.10–1.37)	< 0.001
≥500	1900	1581 (8.0)	141 (0.7)	178 (0.9)	0.96 (0.84–1.09)	0.520	1.26(1.10–1.45)	0.001
Missing	198	151 (0.8)	24 (0.1)	23 (0.1)	1.48 (1.06–2.06)	0.020	1.15(0.79–1.68)	0.468

Note: ART refers to Antiretroviral Therapy.

The persistence of high-risk profiles suggests that specific subgroups may bypass macro-level policy gains. To determine how these risks translate into actual transmission, we performed longitudinal HIV genetic networks analysis to pinpoint the molecular super-spreaders (“Who”) that function as the primary drivers of the epidemic.

### Topological evolution of the longitudinal HIV genetic networks

**Figure 1.**
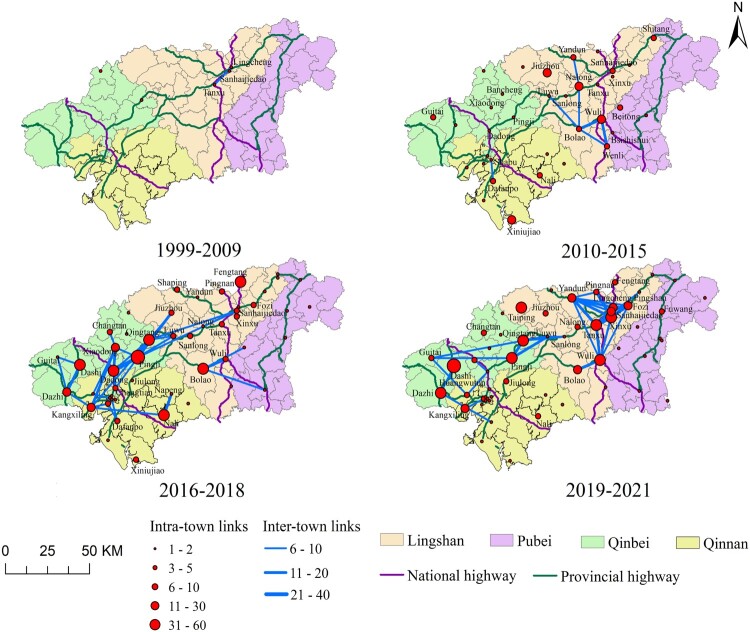
Spatiotemporal visualization of HIV transmission networks in Qinzhou (1999–2021). Township nodes (red circles) are sized by intra-link volume; connecting lines (blue) represent inter-link strength, weighted by thickness. Transportation routes shown as green (provincial highways) and dark purple (national highways) lines. Administrative regions (districts or counties) colour-coded: Lingshan (beige), Pubei (lavender), Qinbei (light green), Qinnan (mustard).

Based on the 5094-sequence dataset, the topological characteristics of the HIV genetic networks exhibited significant expansion and structural shifts across the four epidemiological phases (see Table 3). The network expanded from 34 nodes and 51 edges (1999–2009) to 729 nodes and 1408 edges (2019–2021). While the average degree peaked at 4.21 in 2016–2018, network density dropped from 0.0909 to 0.0053, signalling a transition from compact early clusters to a sparse, large-scale system. The number of clusters grew 43-fold (from 4 to 175). Notably, the maximum cluster size peaked at 51 in 2016–2018, suggesting this period was a critical window for the formation of major transmission chains. The number of molecular super-spreaders rose from 1 to a peak of 41 in 2016–2018. Although the proportion of molecular super-spreaders declined to 5.35% by the final phase, their edge proportion reached a record high of 61.65%. This indicated that a decreasing minority of individuals was responsible for an increasing majority of transmission links. The HIV genetic networks across the four epidemiological phases are shown in Supplementary file 1 Figure S4, and their geographic distributions are presented in Figure 1.

**Table 3.** HIV genetic network metrics across four distinct epidemiological phases (*N* = 5094).

Phase	No. of nodes	No. of edges	Network density	Average degree	Average degree centrality	Average betweenness	Average eigenvector centrality	Maximum degree centrality
1999–2009	34	51	0.0909	3.00	0.0909	0.032475	0.2303	0.5455
2010–2015	328	548	0.0102	3.34	0.0102	0.000152	0.0452	0.0550
2016–2018	627	1321	0.0067	4.21	0.0067	0.000033	0.0300	0.0431
2019–2021	729	1408	0.0053	3.86	0.0053	0.000017	0.0347	0.0467

Phase	Maximum betweenness	Maximum eigenvector centrality	No. of super spreaders	Total Edge Count of Super spreaders	Proportion of Super spreaders	Number of clusters	Maximum cluster size	Average Cluster Size	Edge Proportion of Super spreaders
1999–2009	0.4054	1	1	18	0.0294	4	28	8.5	0.35294118
2010–2015	0.0070	1	26	319	0.0793	69	37	4.75	0.58211679
2016–2018	0.0023	1	41	764	0.0654	148	51	4.24	0.57834974
2019–2021	0.0017	1	39	868	0.0535	175	36	4.17	0.61647727

### Profiles of molecular super-spreaders

Based on HIV genetic networks constructed from all 5094 available sequences, Table 4 summarizes the profiles of identified molecular super-spreaders across the four phases. The number of super-spreaders increased from 1 to 39, with their maximum degree centrality rising from 18 to 34. The transmission dynamics shifted from younger IDU populations in the early stages to an older, heterosexually active demographic in the final phase. Although men dominated, the composition of women increased from 0.0% in phase 1 to 20.5% in phase 4. By 2019–2021, 94.9% of molecular super-spreaders were ≥50 years old. Within this group, farmers accounted for 82.1% and commercial heterosexual contact for 71.8% of cases. The subtype distribution shifted from an exclusive CRF08_BC presence (100.0%) in Phase 1 to a more diverse mixture in subsequent phases, eventually being overtaken by CRF07_BC (74.4%) in Phase 4. Geographically, molecular super-spreaders evolved from a localized focus in TX township to a multi-focal distribution, anchored in townships along major transportation hubs, such as the townships of PJ, QT, and XX. While local ART coverage increased from 56.1% in 2016 to 86.7% by 2021, molecular super-spreaders exhibited a concurrent rise in dropout rates, escalating from 19.2% in Phase 2 to 28.2% in Phase 4. Furthermore, the proportion of these super-spreaders with unsuppressed (≥ 200 copies/mL) or missing viral loads rose from 0.0% in Phase 2 to 38.5% by Phase 4, indicating a marked decline in treatment adherence within the identified transmission networks.

**Table 4.** Spatiotemporal profiles and epidemiological characteristics of molecular super-spreaders across four distinct phases (1999–2021) (*N* = 5094).

Network Indicators	Phase 1	Phase 2	Phase 3	Phase 4
	1999–2009	2010–2015	2016–2018	2019–2021
Number of super spreaders	1	26	41	39
Male (*n*, %)	1 (100.0)	23 (88.5)	36 (87.8)	31 (79.5)
Mean Degree Centrality	18.0	12.3	18.6	22.3
Maximum degree Centrality	18	18	27	34
Demographics				
Age	< 25 (100.0%)	50–69 (50.0%); 35–49 (26.9%); ≥ 70: (19.2%); 25–34: (3.8%)	50–69 (65.9%); ≥ 70 (17.1%); 35–49 (17.1%)	50–69 (59.0%); ≥ 70 (35.9%); 35–49 (5.1%)
Occupation	Farmer (100.0%)	Farmer (80.8%); House worker or business (15.4%); Other (3.8%)	Farmer (85.4%); Retired (9.8%); House worker or business (4.9%)	Farmer (82.1%); House worker or business (15.4%); Retired (2.6%)
Marital status	Single (100.0%)	Married (53.8%); Divorced or widowed (26.9%); Single (19.2%)	Married (73.2%); Divorced or widowed (19.5%); Single (7.3%)	Married (59%); Divorced or widowed (30.8%); Single (10.3%)
Risk Factors				
Transmission route	IDU (100%)	Commercial (42.3%); Non-marital heterosexual (23.1%); IDU: 15.4%; Non-marital non-commercial (15.4%); Spouse positive (3.8%)	Commercial (82.9%); Spouse positive (9.8%); Non-marital non-commercia (7.3%)	Commercial (71.8%); Non-marital non-commercia (23.1%); Spouse positive (5.1%)
ART* and Virological Profile				
Active ART	0 (0.0%)	12 (46.2%)	6 (14.6%)	7 (17.9%)
ART dropout	1 (100.0%)	5 (19.2%)	10 (24.4%)	11 (28.2%)
Active ART but				
1. Viral load ≥ 200 copies/mL	0 (0.0%)	0 (0.0%)	4 (9.8%)	1 (2.6%)
2. Viral load missing	1 (100.0%)	0 (0.0%)	6 (14.6%)	14 (35.9%)
Dominant HIV subtype	CRF08_BC (100%)	Other (69.2%); CRF01_AE_C2 (26.9%); CRF08_BC (3.8%)	CRF08_BC (43.9%); CRF07_BC (19.5%); Other (19.5%); CRF01_AE_C2 (17.1%)	CRF07_BC (74.4%); CRF08_BC (23.1%); CRF01_AE_C2 (2.6%)
Geographic Focus				
Key Townships	TX: 100%	BL: 12%; JS: 8%; KXL: 8%; WL1: 8%; JZ: 8%; WL2: 8%; NL: 8%; SH: 4%; DZ: 4%; WF: 4%; XX: 4%; TX: 4%; LC: 4%; CT1: 4%; LW: 4%; QT: 4%; HWT: 4%	PJ: 19.5%; QT: 17.1%; NP: 4.9%; LW: 4.9%; SH: 2.4%; SL: 2.4%; DC: 2.4%; BT: 2.4%; XY: 2.4%; DD: 2.4%; DS: 2.4%; DZ: 2.4%; ZC: 2.4%; AS: 2.4%; XD: 2.4%; JS: 2.4%; KXL: 2.4%; WL1: 2.4%; JZ: 2.4%; TX: 2.4%; SB: 2.4%; GT: 2.4%; NL: 2.4%; HT: 2.4%; HWT: 2.4%; LM: 2.4%	TX: 12.8%; XX: 10.3%; LC: 10.3%; YD: 10.3%; FZ: 7.7%; SH: 5.1%; PJ: 5.1%; WL2: 5.1%; CT2: 5.1%; DD: 2.6%; DS: 2.6%; ZC: 2.6%; XJ: 2.6%; PN: 2.6%; PS: 2.6%; XNJ 2.6%; FW: 2.6%; NL: 2.6%; QT: 2.6%; HWT: 2.6%

Note: Super spreader was defined as its degree centrality exceeded the threshold of two standard deviations above the mean (*μ* + 2*σ*) for each respective phase.

*ART denotes antiretroviral therapy.

To investigate how these individual-level transmission risks scale up to shape the broader regional epidemic, we further conducted phylodynamic and viral migration analyses across demographic subgroups.

### Phylodynamic reconstruction and demographic transmission dynamics

#### Bayesian evolutionary reconstruction of HIV-1 subtypes/clusters

Bayesian phylodynamic analysis revealed that five HIV-1 subtypes independently converged into a shared heterosexual-driven transmission network in Qinzhou. CRF01_AE (Cluster 1 and 2) (Supplementary file 1 Figure S3-1 and S3-2) and CRF08_BC (Supplementary file 1 Figure S3-4) originated from regional IDUs in the mid-1990s before transitioning to heterosexual transmission circa 1999. CRF07_BC (Supplementary file 1 Figure S3-3), initially circulating among men who have sex with men (MSM), later diverged into distinct heterosexual and MSM-hybrid clusters. While CRF55_01B (Supplementary file 1 Figure S3-5) predominantly affected younger men after 2005, other major lineages exhibited a progressive convergence towards older age groups, establishing a spatiotemporally overlapping transmission reservoir among the rural elderly. Time to most recent common ancestor (tMRCA) is shown in Supplementary file 1 Table S5, all major estimates had strong statistical support, posterior probability (PP) ≥ 0.90.

#### Temporal dynamics of lineage composition defined by age-gender state frequencies

**Figure 2.**
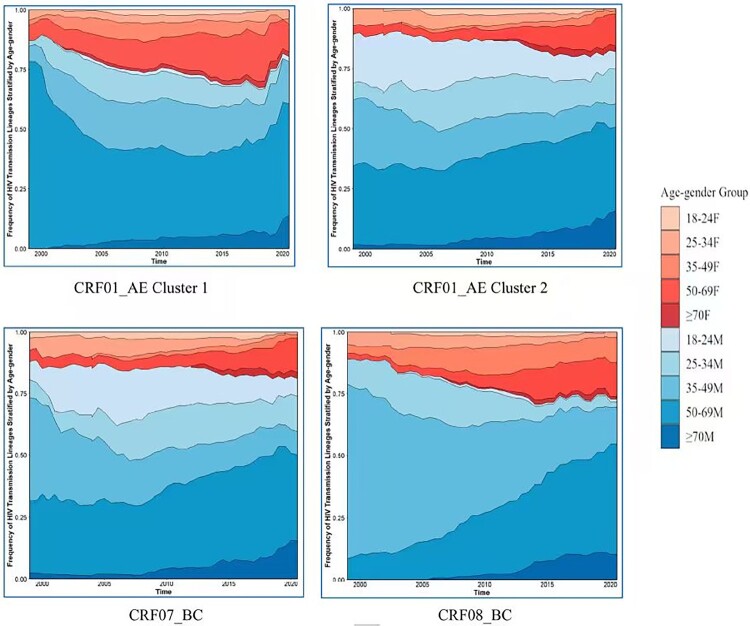
Temporal dynamics of lineage composition based on age-gender state frequencies for major HIV-1 lineages. F, female; M, male. Continuous temporal mapping of ancestral age-gender traits was performed using a Bayesian phylogeographic framework. State frequencies were estimated using discrete trait ancestral reconstruction, representing the posterior probability distribution of age-gender attributes across the reconstructed phylogenetic trees. The Y-axis represents the relative contribution of each age-gender group to the total Markov jump events at a given time point. Each panel represents a specific HIV-1 lineage (subtype/cluster), showing the proportional contribution of ten demographic groups (stratified by age and gender) to the regional epidemic pool over time. A notable expansion in the proportion of older males (50–69M) is observed in later years within CRF01_AE Cluster 2, CRF07_BC, and CRF08_BC.

The smoothed stacked area charts (Figure 2) delineate the temporal shifts in lineage composition across four epidemiological phases. By quantifying reconstructed ancestral states, these visualizations reveal a progressive demographic shift, as the 18–49 age group contracted, older cohorts (particularly those aged 50–69 and ≥70) expanded to become the predominant components across all major lineages. This transition was particularly aggressive in CRF08_BC, where the older cohort’s contribution surged from negligible levels in the early 2000s to dominance by 2008. While CRF01_AE Cluster 1 maintained long-standing geriatric dominance, CRF01_AE Cluster 2 and CRF07_BC underwent a steady replacement of middle-aged males by older cohorts, a shift that accelerated after 2010. Male lineages consistently predominated the transmission pool. By 2015, these convergent trajectories had established the rural male elderly as the primary and expanding transmission reservoir for the regional HIV-1 flow.

#### Inference of asymmetric transmission dynamics between age-gender subgroups

Subsequently, BSSVS analysis (Supplementary Table S6, Figure S5) identified bidirectional and asymmetrical viral flows across age-gender cohorts. The 50–69M group exhibited the highest outward transition rates across all lineages, acting as the predominant source of viral dissemination. Specifically, in CRF01_AE Cluster 2, robust transmissions were observed from 50–69M to females to nearly all age groups (BF = 670,332), alongside significant internal transmission (50–69M → 50–69F). In contrast, the 35–49M group primarily facilitated both intra-group transmission and downward viral flow towards younger cohorts, notably via the 35–49M → 35–49M and 35–49M → 25–34F pathways, respectively, across all lineages except for CRF08_BC. While the lineage state frequency of the ≥70 group increased over time (Figure 2), BSSVS results characterized this cohort as a net recipient of infections. Transitions into the ≥70 group from the 50–69M reservoir were highly supported, yet outward transitions from the ≥70 group remained minimal across all lineages.

### Coupled spatiotemporal evolution of HIV genetic networks with transportation infrastructure

Following the identification of “Who” drives the epidemic, we characterized the “Where” by integrating longitudinal genetic network metrics with spatial statistical analysis. The spatial distribution of network connectivity (represented by mean degree centrality) showed a progressive geographical dispersion over the study period. This trend was evidenced by the Global Moran’s I, demonstrated in Supplementary file 1 Table S7, which declined from 0.298 (1999–2009, *P* < 0.001) to a non-significant 0.115 by 2019–2021 (*P* = 0.057). In contrast, recent network expansion, measured by MMCG, exhibited an opposing trend of increasing spatial concentration. The clustering of MMCG remained consistently significant (*P* < 0.01) and peaked during the 2019–2021 period (I = 0.373, Z = 4.765), indicating that recent transmission hotspots have become increasingly localized.

**Figure 3.**
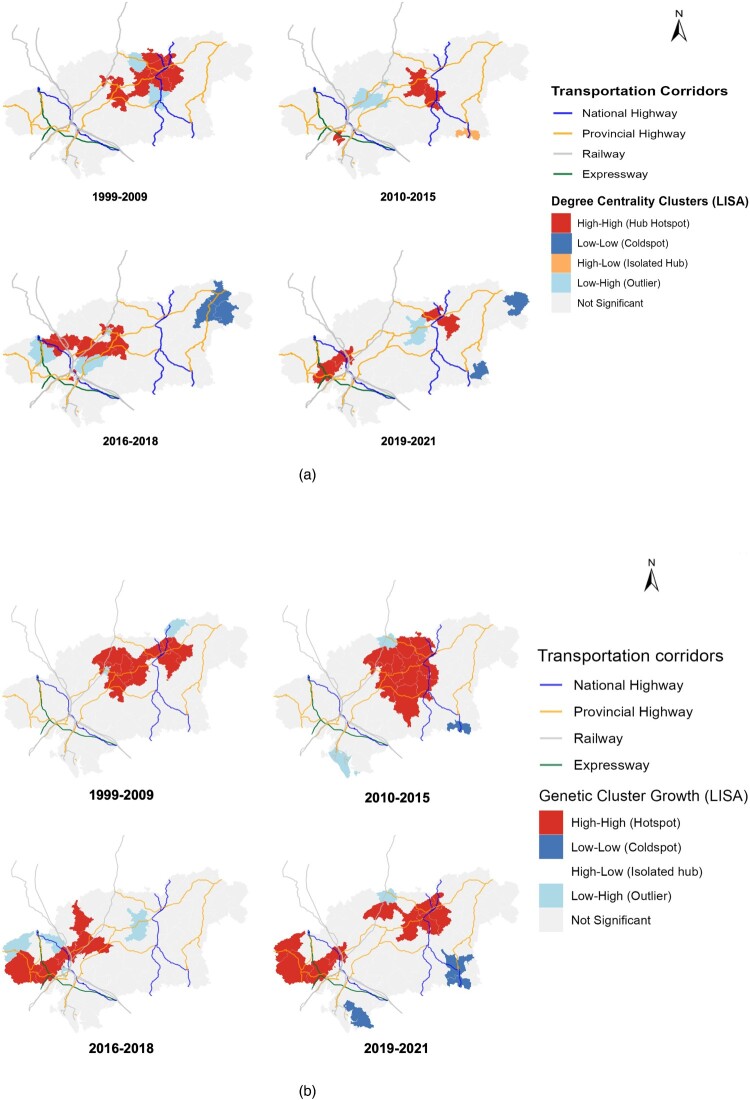
(a) Spatiotemporal local indicators of spatial association analysis of the association between HIV genetic network degree centrality and transportation corridors (1999–2021). (b) Spatiotemporal local indicators of spatial association analysis of the association between HIV genetic cluster growth and transportation corridors (1999–2021).

Local indicators of spatial association (LISA) analysis demonstrated a significant geographic alignment between HIV expansion and transportation arteries. For historical connectivity (Figure 3(a)), High-High hubs of degree centrality were initially concentrated at major highway arteries in the East-north region (1999–2009). Over the 23-year period, these connectivity hubs progressively migrated along the national and provincial highway arteries and established new transmission clusters in western areas by 2019–2021. Parallel LISA analysis of MMCG (Figure 3(b)) confirmed that recent genetic growth followed a similar trajectory but with higher intensity. Recent hotspots were consistently anchored to and extended along the primary road networks, with genetic expansion mirroring the layout of national (blue lines) and provincial (orange lines) highway arteries.

**Figure 4.**
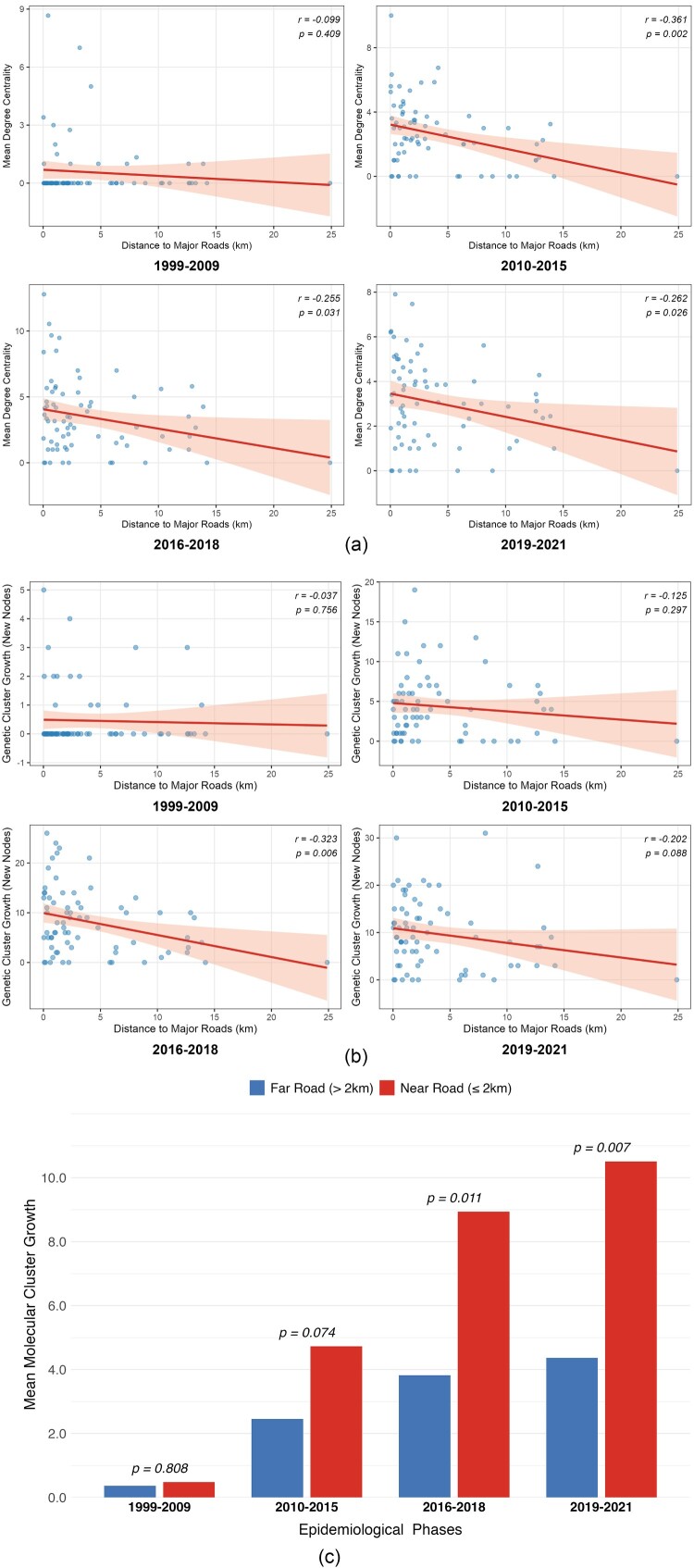
(a) Correlation analysis of HIV genetic network degree centrality and geographic proximity to transportation network. (b) Correlation analysis of HIV genetic network growth and geographic proximity to transportation network. (c) Comparison of mean HIV molecular cluster growth between townships within and outside the 2-km transportation buffer zones.

Distance-base quantitative analysis further substantiated the role of transportation corridors as primary conduits for viral expansion. Correlation analysis identified a negative relationship between geographic proximity to roads and genetic network metrics (Figure 4(a,b)). For historical connectivity, the correlation with road distance became significant after 2010 (2010–2015: *r* = −0.361, *P* = 0.002), while genetic growth peaked in its dependency on road proximity during 2016–2018 (*r* = −0.323, *P* = 0.006). Crucially, transportation buffer zone analysis (Figure 4(c)) revealed a temporal emergence of stark divergence in transmission intensity based on road proximity. While no significant difference was observed in the earliest phase (1999–2009, *P* = 0.808), townships located within 2-km buffer zones of major roads exhibited significantly higher molecular cluster growth compared to those in distal areas from 2016 onwards (2016–2018, *P* = 0.011; 2019–2021, *P* = 0.007). By the final period (2019–2021), the MMCG in near-road townships was more than double that of distal areas, confirming that the recent intensification of HIV transmission was predominantly concentrated along major transportation arteries. After adjusting for population size and density, the negative binomial regression model reveals a significant independent association between road proximity and the burden of cumulative reported HIV cases [adjusted Incidence Rate Ratio (IRR) = 1.431; 95% CI: 1.143–1.782, *p* = 0.0017]. Furthermore, while townships within the 2-km highway buffer account for 77.9% (2,710,918/3,479,690) of the total population in Qinzhou, they host a disproportionately high 94.4% (101/107) of the identified molecular super-spreaders (*X* ^2^ = 17.65, *P* < 0.001). These findings confirm that the transportation corridor effect persists independently of demographic aggregation.

To elucidate the social-behavioural mechanisms (“How”) underlying the localization of the epidemic along transportation arteries, a supplementary exploratory behavioural survey targeting local men was conducted.

### Behavioural drivers of corridor-based viral dispersal

A total of 84 men were recruited; among 22 seeds (diagnosed HIV cases), only two successfully initiated a single wave of recruitment, referring two and three followers, respectively. The remaining participants (*n* = 57) were recruited via venue-based sampling at informal gathering sites frequented by older rural men. The study participants were predominantly male (97.6%) and aged over 50 years (91.7%), with 48.8% of participants being widowed, divorced, or single. Their social and recreational activities primarily occurred in informal outdoor settings, including village entrances for card playing (75.0%), community parks (56.0%), public squares (54.8%), and periodic township markets (47.6%) etc. Regarding sexual risk, 48.8% of respondents reported engaging in casual or commercial heterosexual sex, with 70.7% practicing inconsistent condom use. Notably, 88.9% of commercial sexual encounters occurred in townships within a 2-km buffer of transportation arteries. The median number of commercial partners for those engaging in commercial sexual contacts was 2 (IQR: 2–4). Commercial sex encounters were characterized by low entry costs (low-tier). Specifically, over one-third of respondents (37.5%) reported prices below 50 CNY per transaction, while only 27.1% exceeded 100 CNY. Furthermore, 60.9% of respondents visited FSWs at least semi-annually. While 66.7% of encountered FSWs were from within Qinzhou, over 70.0% of respondents noted that these FSWs were highly mobile, rotating among townships along major transportation arteries to synchronize with local periodic market days. Detailed survey questions and results are demonstrated in Supplementary file 1 Table S8.

## Discussion

The strength of this study lies in its multi-layered analytical framework, which moves beyond isolated methodologies towards a truly integrative molecular epidemiological approach. On a global scale, landmark research such as the Rakai Community Cohort Study in Uganda [29,30] has demonstrated the power of coupling longitudinal cohorts with deep-sequencing phylogenetics to track transmission bottlenecks. However, our framework extends this paradigm by integrating fine-grained demographic stratification with transportation buffer zone analysis, which is often overlooked in traditional phylodynamic studies. While recent advancements in China have begun to combine genetic networks with spatial mapping [19], they frequently remain descriptive and static. In contrast, our approach uniquely bridges the gap between molecular evidence and structural drivers. By triangulating longitudinal network evolution and phylogeographic reconstruction with transportation-buffer analysis, we identify that the “transportation corridor effect” represents an independent risk factor for the local HIV-1 burden. This finding was further substantiated by the “double-proximity” model identified through our supplementary behavioural survey. This model characterizes the localized, nearby mobility of older men (clients) to townships along the transportation arteries on market days, and the linear mobility of FSWs navigating these corridors to access client flow. Such a holistic “Who-Where-How” characterization provides the granular precision necessary to uncover undiagnosed cases and high-risk individuals within “silent reservoirs,” a depth of insight that macro-level phylogeographic study [20] or purely genetic “snapshots” [15–18] cannot achieve. This study's finding can further facilitate geographically targeted screening and enhanced case-finding along transportation arteries.

The GEE analysis confirmed the significant population-level success of the “Treat All” policy, with active ART reducing secondary transmission risk by 21%. This aligned with the “Treatment as Prevention” (TasP) paradigm, suggesting that early treatment had successfully blunted the overall epidemic trajectory [31]. Regarding the global “95-95-95” targets, China reached an estimated “84-93-97” in 2022 [32]. Our findings suggest that a primary driver of this “first 95” shortfall in rural Southwest China is the under-detection and subsequent “invisibility” of high-risk marginalized groups in genetic networks. Specifically, while the sustained male dominance underscores older men as primary epidemic drivers, this pattern may be biased by the under-sampling of populations such as low-tier FSWs, as indicated in our behavioural survey and previous study [33]. Due to the clandestine nature of their work, high mobility, and significant healthcare barriers [34], these potential core transmitters often remain undiagnosed, leading to their underrepresentation in network analysis. Nevertheless, the rising female proportion among molecular super-spreaders (20.5% in Phase 4) signals an intensifying heterosexual transmission dynamic, further emphasizing the urgent need to bridge this detection gap through geographically and demographically targeted strategies. Furthermore, we identified a 28.2% ART dropout rate and 38.5% rate of unsuppressed or missing viral loads among Phase 4 molecular super-spreaders, reflecting a structural breakdown between the “second” and “third” 95–95–95 targets. The lack of protective effect in dropouts (AOR = 1.01) further underscored that high nominal coverage often masks functional gaps in transmission hubs. In these settings, suboptimal adherence acts as a bottleneck, undermining ART’s capacity to achieve the sustained viral suppression required to break the chain of transmission, a finding consistent with previous studies [35].

Our phylogeographic analysis indicated that the current HIV epidemic in Qinzhou has evolved from isolated outbreaks among IDUs into a mature epidemic fuelled by external viral introductions and subsequent sustained local circulation among older heterosexual populations. This shift is typified by a profound geriatric transition across all viral lineages. As shown in our temporal reconstructions, the contraction of younger cohorts (25–49 years) has led to the epidemic being increasingly sequestered within the oldest demographics, with males aged ≥ 50 emerging as the predominant state. This demographic “takeover,” most aggressive in CRF08_BC and accelerating after 2010 in CRF07_BC, is further corroborated by our identification of molecular super-spreaders within these age groups.

Such a transition aligns with the broader “graying” of China’s HIV epidemic [36,37], a trend particularly pronounced in Qinzhou, which consistently ranked among the top five prefectures in China for new HIV diagnoses among elderly (≥ 50 years) individuals linked to commercial sex [38]. The BSSVS analysis elucidates the functional hierarchy driving this shift. Specifically, males aged 50–69 function as the primary hub of viral dissemination, driving infections both internally and upward to the oldest-old, a phenomenon likely facilitated by low-tier FSWs [39]. These results highlight a critical surveillance gap in rural commercial sex markets [40]. Conversely, despite their declining share in lineage composition, males aged 35–49 act as both intra-group transmission and downward bridges to younger females. This role mirrors patterns observed in other generalized epidemics and ensures the continuous re-seeding and long-term persistence of the regional viral pool [17]. Finally, although the ≥ 70 group is expanding rapidly, it functions predominantly as a transmission sink, absorbing infections from the male aged 50–69 reservoir with minimal outward spread [41]. Collectively, these findings pinpoint the “silent reservoirs” sustaining the regional viral flow, addressing the “Who” in the epidemic’s transmission dynamics.

Our longitudinal genetic network and spatiotemporal analyses reveal a strategic transition of the epidemic from a localized cluster in Tanxu (Phase 1) to a broad geographic concentration anchored along major transportation arteries (“Where”). This spatial shift is underscored by the LISA analysis, which confirms genetic network growth along the transportation corridors, and the persistent impact of transit proximity, evidenced by a 43.1% increased risk of cumulative HIV cases in townships within 2 km buffer of highway arteries. Furthermore, the gap between the 94.4% molecular super-spreader concentration and the 77.9% population baseline confirms that transportation infrastructure acts as an independent functional driver. Rather than a mere proxy for population aggregation, these arteries serve as structural conduits that facilitate and anchor regional viral dispersal. This “corridor-driven” expansion mirrors the historical dissemination of the HIV-1 primordial ancestor from Kinshasa, which was similarly facilitated by colonial transportation networks [27].

To explain these dynamics, we propose a “double-proximity” model, which integrates supplementary exploratory behavioural survey data with molecular and spatial findings to characterize the spatiotemporal synchronization of high-risk behaviours (“How”). This model is defined by the convergence of two distinct mobility patterns: the localized mobility of clients and the linear mobility of providers. Specifically, older rural males (≥ 50 years) exhibit restricted, routine-driven mobility, seeking low-tier commercial sex at township hubs along the transportation arteries during periodic market days. Conversely, low-tier FSWs exhibit linear, corridor-based mobility, rotating among venues along highway axes to access these periodic market-day client flows [42]. This dual alignment of geographic proximity and behavioural timing creates transient, high-intensity “risk hubs” that facilitate the sustained dispersal of HIV-1 lineages across the regional network. This infrastructure-dependent pattern diverges from the long-distance migratory models seen in Sub-Saharan Africa [43] or urban MSM-driven epidemics in China [44]. Instead, it highlights how transportation corridors serve as the primary conduits for viral spread among an aging, non-migratory population.

Hence, future interventions should shift from passive surveillance to data-driven proactive engagement. Outreach must prioritize MMCG hotspots along transportation corridors to optimize resource allocation. Specifically, mobile outreach taskforces should provide point-of-care testing directly at clandestine venues, such as roadside inns, rental houses, and informal gathering spots, where elderly individuals and FSWs congregate. To normalize screening and reduce stigma, HIV testing should be integrated into routine geriatric physical exams at township hospitals or health centres under China’s basic public health services. For identified cases, community-based follow-up models involving village doctors were essential to provide tailored adherence support and reduce high dropout rates among elderly cases especially for those molecular super-spreaders [45]. Finally, sustained prevention efforts, including peer education and venue-based outreach at village gathering spots for the elderly, remain crucial for controlling localized outbreaks.

This study has several limitations. First, as the sample is from only one prefecture in Guangxi, it may not fully represent the entire southwest of China. However, since Qinzhou shares similar HIV/AIDS epidemic trends and characteristics with Guangxi, the findings still hold significance and can guide precise intervention. Second, while the supplementary behavioural survey had a limited sample size and potential selection bias due to venue-based sampling, it was designed as a contextual probe rather than a large-scale representative study. These findings provide critical “ground-truth” for the socio-behavioural mechanisms driving transmission along transportation arteries. While not fully generalizable provincial-wide, they offer essential mechanistic insights into the localized dynamics of the epidemic. Third, the number of molecular super-spreaders may be underestimated due to our stringent identification threshold (μ + 2σ) and the inherent exclusion of undiagnosed or unsequenced individuals. While such sampling gaps, a common challenge in molecular epidemiology, could omit certain high-risk groups like low-tier FSWs; this longitudinal dataset remains the most representative empirical evidence currently available for characterizing regional transmission dynamics. Finally, while the link between road proximity and HIV burden was independent of population metrics; unmeasured socioeconomic confounders like gross domestic product or urbanization may persist. Future research incorporating multidimensional socioeconomic data is needed to isolate the specific effects of infrastructure from regional development.

## Supplementary Material

Supplemntary file 2 20260422.docx

Supplementary file 1 20260422.docx

## Data Availability

The HIV sequences and related epidemiological data used in this study were collected by our team from Qinzhou, Guangxi, China. These data and related datasets are not publicly available but can be obtained upon reasonable request and approval from the Chinese Center for Disease Control and Prevention. Requests for accessing these data and datasets should be directed to YR via email: ruanyuhua92@chinaaids.cn.
