# Supplementary material for: Delineating the “who-where-how” of persistent HIV epidemics: a 23-year longitudinal genetic network, phylodynamic, and spatial analysis for precise intervention in rural China
Source: Emerg Microbes Infect. 2026 May 13;15(1):2671470. doi: 10.1080/22221751.2026.2671470 (PMC13224696; doi:10.1080/22221751.2026.2671470)
Supplement: Supplemntary file 2 20260422.docx [file TEMI_A_2671470_SM8352.docx]

Supplementary file 2

Methods for details

1. Stratification of four epidemiological phases

The 23-year study period (1999-2021) was stratified into four distinct epidemiological phases based on key public health milestones and policy shifts.

Phase I [1999-2009, Pre-Guangxi AIDS Conquering Project (GACP) phase]: This period represents a stage of natural epidemic growth characterized by the implementation of routine prevention and control measures. During this phase, the epidemic expanded without the intensive, large-scale interventions that were later introduced under the GACP, providing a baseline of viral transmission dynamics in the region.

Phase II (2010-2015, GACP intensive intervention phase): This phase is marked by the large-scale implementation of regional prevention and control measures under the GACP, aiming to curb the rapidly rising epidemic.

Phase III (2016-2018, “Treat All” transition phase): Although the national “Treat All” policy was officially initiated in 2016, its comprehensive coverage in rural Southwest China was progressively phased in. This period represents the transition from targeted clinical eligibility to universal access in resource-limited settings.

Phase IV (2019-2021, “Treat All” full coverage phase): This phase is defined by the stabilized and comprehensive implementation of the “Treat All” strategy across the rural study areas, representing the contemporary management era.

1. Phylodynamic and phylogeographic analysis

2.1 Sequence preparation and subsampling

HIV-1 sequences were edited and aligned using BioEdit (v7.0.9.0) and the HIV Align tool. Subtypes and clusters were identified via phylogenetic reconstruction in MEGA (v10.0). For the two major subtype/clusters exceeding 1,000 sequences (CRF01_AE Cluster 2 and CRF08_BC), we performed stratified random subsampling (n=500 each) to ensure Bayesian computational feasibility while preserving demographic representativeness (Supplementary file 1 Table S2). To enhance molecular clock calibration, 61 high-quality historical reference sequences (sampled 1997-2013) from the provincial HIV surveillance database of Guangxi Center for Disease Control and Prevention were integrated to corresponding subtype/cluster dataset as temporal anchors to estimate the Time of the Most Recent Common Ancestor (tMRCA) for each subtype/cluster (Supplementary file 1 Table S3).

2.2 Bayesian evolutionary reconstruction of HIV-1 subtypes/clusters

Phylodynamic analyses were conducted in BEAST (v1.8.4) for five lineages: CRF01_AE (Clusters 1 & 2), CRF07_BC, CRF08_BC, and CRF55_01B. Other lineages were excluded from these analyses due to insufficient sample sizes for robust inference. We employed a General Time-Reversible (GTR) substitution model, a relaxed uncorrelated lognormal (UCLN) molecular clock, and a Skygrid demographic prior ^[1–4]^. Markov Chain Monte Carlo (MCMC) chains were run for 1 billion iterations, with the initial 10-30% discarded as burn-in. Convergence was confirmed using Tracer (v1.6), ensuring all parameters achieved an Effective Sample Size (ESS) >200 ^[5]^. Maximum Clade Credibility (MCC) trees were generated via TreeAnnotator and visualized in FigTree (v1.4.4).

- 1. Temporal composition and migration dynamics of viral lineages

Due to sample size constraints, this analysis focused on four predominant HIV-1 lineages (CRF01_AE clusters 1 and 2, CRF07_BC, and CRF08_BC), excluding the less prevalent CRF55_01B.

1. Temporal dynamics of lineage composition defined by age-gender state frequencies: To characterize the temporal evolution of viral lineage distributions across different demographic (age-gender) subgroups, we extracted lineage state frequencies from the annotated MCC trees. Following established phylodynamic visualization protocols ^[1]^, we performed time-slicing across the phylogeny at 0.5-year intervals from the root to the most recent sampling date. This approach allowed us to quantify the temporal shifts in lineage composition and capture how the relative proportions of each age-gender group within the regional viral pool evolved over the 23-year period. At each discrete time point, we identified all co-existing viral lineages and extracted their most probable reconstructed age-gender ancestral states. The relative proportions of lineages belonging to each demographic group were then visualized using smoothed stacked area charts, implemented programmatically via the treeio ^[10]^ and ggplot2 R packages.

(2) Inference of asymmetric transmission dynamics between age-gender subgroups: The migration dynamics and transmission pathways between age-gender subgroups were reconstructed using the Bayesian Stochastic Search Variable Selection (BSSVS) framework ^[6]^, complemented by Markov jump analysis to quantify the directionality and intensity of viral spread throughout the study period ^[7]^. The statistical robustness of the directional transmission pathways between age-gender subgroups was evaluated over the entire 1999-2021 period using Bayes Factors (BF) and posterior probabilities calculated via SpreaD3 v0.9.7.1 ^[8]^. Following the standard interpretation scales, BF values are categorized as follows: 10 ≤ BF < 100 indicates strong evidence and BF ≥ 100 indicates decisive evidence ^[9]^. To ensure the highest level of inferential stringency and minimize spurious linkages within our complex transmission network, we adopted a highly conservative threshold of BF ≥ 100,000 and posterior probability ≥ 0.9.

1. Mean Molecular Cluster Growth calculation

The localized expansion intensity was quantified as Mean Molecular Cluster Growth (MMCG). For a given township *j* at stage *t*, MMCG is defined as:

MMCG *_j,t_* = $\frac{\sum_{vi\in Vt} 1(vi \in\mathrm{Township}j \wedge vi \notin Vt-1)}{Nj}$.

Parameter Annotation: *V_t_*, *V_t-1_*: Sets of nodes in the genetic network at stage *t* and *t-1*, respectively.

1(·): An indicator function identifying “growth nodes” (incident cases) geographically located within township *j*.

*N_j_*: The normalization factor. In this study, *N_j_* = 1 was applied to quantify the absolute expansion magnitude as a proxy for transmission intensity, this approach emphasizes total viral lineage influx along key transportation corridors and eliminates inaccuracies associated with transient population denominators in rural areas.

Zero-filling: Townships with no incident cases were assigned a value of 0 to ensure a continuous surface for subsequent spatial modeling.

1. Spatial autocorrelation analysis

Temporal shifts in spatial clustering of mean degree centrality and MMCG were monitored using the Global Moran’s I index at each phase. To identify geographic hotspots, Local Indicators of Spatial Association (LISA) analysis ^[12]^ based on Local Moran’s I was performed for both HIV genetic network mean degree centrality and MMCG. A Queen’s contiguity weight matrix was used to categorize townships into High-High (hotspots), Low-Low (cold spots), and spatial outliers (High-Low and Low-High). Statistical significance was verified using a Z-test based on asymptotic normal distribution.

1. Road proximity analysis

We calculated the minimum geodesic distance from each township’s geometric centroid to the nearest segment of the regional transportation network (comprising expressways, national and provincial highways) using the sf package in R. Pearson correlation coefficients (r) were employed to quantify the association of both township-level mean degree centrality and MMCG with road proximity. For categorical comparison, townships were stratified into “Near-road” (≤2 km) and “Far-road” (> 2 km) groups using a spatial buffer. Disparities in MMCG between townships with “Near-road” (≤ 2 km) and “Far-road” (> 2 km) were validated using the Wilcoxon rank-sum test. To isolate the impact of road proximity from the effects of general population aggregation, we performed a Negative Binomial regression analysis. While the model used cumulative reported HIV case counts as the outcome, the inclusion of a log(Total_Pop) offset term effectively modeled the cumulative incidence across townships. This framework was specifically chosen to address the overdispersion inherent in the count data and the potential confounding by demographic clustering. The formula was specified as: log(μ) = *β*_0_ + *β*_1_* Road_Proximity + *β*_2_ *Pop_Density + log(Total_Pop), where μ represents the number of cumulative reported HIV cases.

1. Generalized estimating equations analysis

A Generalized Estimating Equations (GEE) model was used to assess the association between ART status and onward HIV transmission among 5,094 HIV/AIDS cases diagnosed between 1999 and 2021. To evaluate the “Treat All” policy, we constructed a dynamic transmission reservoir that yielded 19,661 observations. These observations capture the ART status of potential sources at the end of each year *t* (2016-2020), with each status linked to the occurrence of new molecular linkages in the subsequent year *t+1* (2017–2021) ^[13]^. This lagged design ensures that the identified drivers (ART status) precede the transmission events in time. The reservoir was maintained as follows: (1) individuals diagnosed between 1999 and 2016 (excluding those deceased by the end of 2016) formed the initial baseline pool; (2) cases newly diagnosed between 2017 and 2020 were sequentially incorporated into the pool in the year following their diagnosis; and (3) individuals were removed annually at the end of the year of their death to maintain an active reservoir. Cases diagnosed in 2021 were used to ascertain transmission outcomes but did not contribute to the observations as potential sources. The ART status was categorized as ART-naïve, active ART, or ART dropout. Within-subject correlations were addressed using an exchangeable correlation matrix ^[14]^. The model was adjusted for socio-demographics, risk, residence, HIV subtype, and baseline CD4 level. A two-tailed P < 0.05 was considered significant.

**List of abbreviations:**

GACP: Guangxi AIDS Conquering Project;

tMRCA: time to the most recent common ancestor;

GTR: general time-reversible;

UCLN: uncorrelated lognormal;

MCMC: Markov chain Monte Carlo;

ESS: Effective Sample Size;

MCC: Maximum clade credibility;

BSSVS: Bayesian stochastic search variable selection;

BF: Bayes Factors;

MMCG: Mean Molecular Cluster Growth;

LISA: Local Indicators of Spatial Association;

GEE: Generalized Estimating Equations;

ART: antiretroviral therapy.

Reference

1. Faria NR, Rambaut A, Suchard MA, Baele G, Bedford T, Ward MJ, *et al.* The early spread and epidemic ignition of HIV-1 in human populations. Science 2014;346:56–61.

2. Drummond AJ, Suchard MA, Xie D, Rambaut A. Bayesian phylogenetics with BEAUti and the BEAST 1.7. Mol Biol Evol 2012;29:1969–73.

3. Gill MS, Lemey P, Faria NR, Rambaut A, Shapiro B, Suchard MA. Improving Bayesian population dynamics inference: a coalescent-based model for multiple loci. Mol Biol Evol 2013;30:713–24.

4. Drummond AJ, Ho SYW, Phillips MJ, Rambaut A. Relaxed phylogenetics and dating with confidence. PLoS Biol 2006;4:e88.

5. Rambaut A, Drummond AJ, Xie D, Baele G, Suchard MA. Posterior Summarization in Bayesian Phylogenetics Using Tracer 1.7. Syst Biol 2018;67:901–4.

6. Lemey P, Rambaut A, Drummond AJ, Suchard MA. Bayesian phylogeography finds its roots. PLoS Comput Biol 2009;5:e1000520.

7. Minin VN, Suchard MA. Counting labeled transitions in continuous-time Markov models of evolution. J Math Biol 2008;56:391–412.

8. Bielejec F, Baele G, Vrancken B, Suchard MA, Rambaut A, Lemey P. SpreaD3: Interactive Visualization of Spatiotemporal History and Trait Evolutionary Processes. Mol Biol Evol 2016;33:2167–9.

9. Kass RE, Raftery AE. Bayes factors. J Am Stat Assoc. 1995;90(430):773–795.

10. Wang L-G, Lam TT-Y, Xu S, Dai Z, Zhou L, Feng T, *et al.* Treeio: An R Package for Phylogenetic Tree Input and Output with Richly Annotated and Associated Data. Mol Biol Evol 2020;37:599–603.

11. Minin VN, Suchard MA. Counting labeled transitions in continuous-time Markov models of evolution. J Math Biol 2007;56:391–412.

12. Moran P a. P. Notes on continuous stochastic phenomena. Biometrika 1950;37:17–23.

13. Chen Y, Cao Z, Li J, Chen J, Zhu Q, Liang S, *et al.* HIV transmission and associated factors under the scale-up of HIV antiretroviral therapy: a population-based longitudinal molecular network study. Virol J 2023;20:289.

14. Ballinger GA. Using Generalized Estimating Equations for Longitudinal Data Analysis. Organ Res Methods 2004;7:127–50.
