# Supplementary material for: Delineating the “who-where-how” of persistent HIV epidemics: a 23-year longitudinal genetic network, phylodynamic, and spatial analysis for precise intervention in rural China
Source: Emerg Microbes Infect. 2026 May 13;15(1):2671470. doi: 10.1080/22221751.2026.2671470 (PMC13224696; doi:10.1080/22221751.2026.2671470)
Supplement: Supplementary file 1 20260422.docx [file TEMI_A_2671470_SM8351.docx]

Supplementary file 1

Table S1 Distribution of HIV subtypes/clusters among study samples with RNA sequence in Qinzhou (1999-2021) (N=4,814)

| Items | Numbers | % |
| --- | --- | --- |
| Total | 4814 | 100.0 |
| HIV subtypes |  |  |
| CRF01_AE | 2505 | 52.1 |
| *cluster 1* | *508* | *10.6* |
| *cluster 2* | *1770* | *36.7* |
| *cluster 3* | *14* | *0.3* |
| *cluster 4* | *13* | *0.3* |
| *cluster 5* | *7* | *0.2* |
| *Other CRF01_AE clusters* | *193* | *4.0* |
| CRF07_BC | 491 | 10.2 |
| CRF08_BC | 1527 | 31.7 |
| CRF55_01B | 49 | 1.0 |
| B | 18 | 0.4 |
| Other CRFs or URFs | 224 | 4.6 |

Note: CRF refers to circulating recombinant form, URF refers to Unique recombinant form.

Table S2 Comparison of baseline characteristics between sampled and overall study participants for CRF01_AE cluster 2 (N=1,770) and CRF08_BC (N=1,527) Subtypes in Qinzhou

|  | CRF01_AE cluster 2 | | |  | CRF_08BC | | |
| --- | --- | --- | --- | --- | --- | --- | --- |
|  | All sequences (N, %) | Samples (N, %) | *P* value |  | All sequences (N, %) | Samples (N, %) | *P* value |
| Total | 1770 (100.0) | 500 (100.0) |  |  | 1527 (100.0) | 500 (100.0) |  |
| Age (years) |  |  |  |  |  |  |  |
| 18-24 | 29 (1.6) | 25 (5.0) | 1.000 |  | 38 (2.5) | 29 (5.8) | 1.000 |
| 25-34 | 213 (12.0) | 75 (15.0) | 1.000 |  | 208 (13.6) | 69 (13.8) | 0.998 |
| 35-49 | 447 (25.3) | 128 (25.6) | 0.992 |  | 549 (35.9) | 185 (37) | 0.992 |
| 50-69 | 856 (48.4) | 203 (40.6) | 0.134 |  | 594 (38.9) | 169 (33.8) | 0.477 |
| ≥70 | 225 (12.7) | 69 (13.8) | 0.999 |  | 138 (9) | 48 (9.6) | 0.999 |
| Gender |  |  |  |  |  |  |  |
| Male | 1329 (75.1) | 369 (73.8) | 0.953 |  | 1121 (73.4) | 368 (73.6) | 0.989 |
| Female | 441 (24.9) | 131 (26.2) | 0.997 |  | 406 (26.6) | 132 (26.4) | 0.982 |
| Education |  |  |  |  |  |  |  |
| Illiteracy | 100 (5.6) | 22 (4.4) | 0.999 |  | 78 (5.1) | 25 (5.0) | 1.000 |
| Elementary school | 930 (52.5) | 247 (49.4) | 0.768 |  | 812 (53.2) | 269 (53.8) | 0.985 |
| Junior school | 617 (34.9) | 203 (40.6) | 1.000 |  | 548 (35.9) | 182 (36.4) | 0.987 |
| High school | 113 (6.9) | 28 (5.6) | 0.999 |  | 89 (5.8) | 24 (4.8) | 0.999 |
| Marital status |  |  |  |  |  |  |  |
| Single | 319 (18.0) | 110 (22.0) | 1.000 |  | 392 (25.7) | 121 (24.2) | 0.945 |
| Married | 1062 (60) | 297 (59.4) | 0.961 |  | 834 (54.6) | 285 (57.0) | 0.998 |
| Divorced/Widowed | 389 (22) | 93 (18.6) | 0.791 |  | 301 (19.7) | 94 (18.8) | 0.978 |
| Occupation |  |  |  |  |  |  |  |
| Farmer | 1329 (75.1) | 355 (71) | 0.656 |  | 1174 (76.9) | 376 (75.2) | 0.932 |
| Other | 441 (24.9) | 145 (29) | 1.000 |  | 353 (23.1) | 124 (24.8) | 0.998 |
| Risk |  |  |  |  |  |  |  |
| Injecting drug use | 39 (2.2) | 39 (7.8) | 1.000 |  | 265 (17.3) | 96 (19.2) | 0.999 |
| Heterosexual | 1727 (97.6) | 457 (91.4) | 0.185 |  | 1262 (82.7) | 404 (80.8) | 0.941 |
| Homosexual | 4 (0.2) | 4 (0.8) | 1.000 |  | 0 (0.0) | 0 (0.0) | - |
| Residence |  |  |  |  |  |  |  |
| Qinnan | 602 (34) | 160 (32) | 0.897 |  | 114 (7.5) | 38 (7.6) | 0.999 |
| Qinbei | 476 (26.9) | 146 (29.2) | 0.999 |  | 238 (15.6) | 74 (14.8) | 0.989 |
| Lingshan | 529 (29.9) | 158 (31.6) | 0.998 |  | 1032 (67.6) | 348 (69.6) | 0.998 |
| Pubei | 145 (8.2) | 34 (6.8) | 0.997 |  | 82 (5.4) | 25 (5.0) | 1.0000 |
| Other | 18 (1.0) | 2 (0.4) | 1.000 |  | 3 (0.2) | 0 (0.0) | - |

Note: Sequences from CRF01_AE cluster 2 and CRF08_BC subtypes were derived from the 4814 - RNA sequence dataset.

Table S3 Distribution of HIV-1 sequences used in the final Bayesian analysis for five HIV-1 subtypes/clusters

| HIV-1 subtype | No. of original study samples* | No. of study samples for Bayesian analysis (Post-subsampling)* | No. of historical reference sequences from Guangxi ^#^ (Year of detection) | Total sample size for Bayesian analysis |
| --- | --- | --- | --- | --- |
| Total | 4345 | 2048 | 61 | 2109 |
| CRF01_AE cluster 1 | 508 | 508 | 10 (2005-2007) | 518 |
| CRF01_AE cluster 2 | 1770 | 500 ^@^ | 27 (1997-2009) | 527 |
| CRF07_BC | 491 | 491 | 12 (2009-2010) | 503 |
| CRF08_BC | 1527 | 500 ^@^ | 8 (1997-2009) | 508 |
| CRF55_01B | 49 | 49 | 4 (2009-2013) | 53 |

Note: * The study samples were derived from the 4814-RNA sequence dataset.

# Historical reference sequences from Guangxi (n=61, sampled 1997-2013) were retrieved from the provincial HIV surveillance database of Guangxi CDC as temporal anchors for MRCA (most recent common ancestor) calibration.

@ For subtype/clusters with more than 1000 original sequences, a stratified random subsampling strategy was applied to select 500 representative sequences to ensure Bayesian computational feasibility while preserving demographic representativeness.

Table S4 Comparison of demographic characteristics between all newly diagnosed HIV/AIDS cases and samples with RNA sequences in Qinzhou (1999-2021)

| Variables | Newly diagnosed cases N (%) | Samples with RNA sequence N (%) | *P -*value |
| --- | --- | --- | --- |
| Total | 7381 (100.0) | 4814 (100.0) |  |
| Age (years) |  |  |  |
| 18-24 | 408 (5.5) | 178 (3.7) | 1.000 |
| 25-34 | 1056 (14.3) | 697 (14.5) | 1.000 |
| 35-49 | 2112 (28.6) | 1389 (28.9) | 1.000 |
| 50-69 | 3133 (42.4) | 2059(42.8) | 1.000 |
| ≥70 | 672 (9.1) | 491 (10.2) | 1.000 |
| Gender |  |  |  |
| Male | 5542 (75.1) | 3588 (74.5) | 1.000 |
| Female | 1839 (24.9) | 1226 (25.5) | 1.000 |
| Education |  |  |  |
| Illiteracy | 360 (4.9) | 232 (4.8) | 1.000 |
| Elementary school | 3791 (51.4) | 2451 (50.9) | 1.000 |
| Junior school | 2534 (34.3) | 1722 (35.8) | 1.000 |
| High school and above | 696 (9.4) | 409 (8.5) | 1.000 |
| Marital status |  |  |  |
| Single | 1831 (24.8) | 1126 (23.4) | 1.000 |
| Married | 4087 (55.4) | 2727 (56.7) | 1.000 |
| Divorced/Widowed | 1463 (19.8) | 961 (20.0) | 1.000 |
| Occupation |  |  |  |
| Farmer | 5521 (74.8) | 3602 (74.8) | 1.000 |
| Other | 1860 (25.2) | 1212 (25.2) | 1.000 |
| Risk |  |  |  |
| Injecting drug | 352 (4.8) | 407 (8.5) | 0.997 |
| Heterosexual | 6675 (90.4) | 4320 (89.7) | 1.000 |
| Homosexual | 222 (3.0) | 87 (1.8) | 1.000 |
| Unkown | 132 (1.8) | 0 (0.0) | - |
| Residence |  |  |  |
| Qinnan | 1156 (15.7) | 1037 (21.5) | 0.117 |
| Qinbei | 1402 (19.0) | 1060 (22.0) | 0.996 |
| Lingshan | 3531 (47.8) | 2129 (44.2) | 1.000 |
| Pubei | 1238 (16.8) | 551 (11.5) | 1.000 |
| Other | 54 (0.7) | 37 (0.8) | 1.000 |

Table S5 The time to most recent common ancestor for main HIV subtypes and clusters in Qinzhou

| HIV subtypes/clusters | Guangxi historical reference sequences ^@^ | |  | Study samples^+^ | | tMRCA^*^- 95%HPD^#^ |
| --- | --- | --- | --- | --- | --- | --- |
|  | Year of detection | No. of samples |  | Year of detection | No. of samples |  |
| Total | - | 61 |  | - | 2048 | - |
| CRF01_AE cluster 1 | 2005-2007 | 10 |  | 2010-2020 | 508 | 1997.9 (1996.1- 1999.9) |
| CRF01_AE cluster 2 | 1997-2009 | 27 |  | 2010-2020 | 500 | 1993.6 (1991.9- 1997.2) |
| CRF07_BC | 2009-2010 | 12 |  | 2011-2020 | 491 | 2000.4 (1993.2- 2002.7) |
| CRF08_BC | 1997-2009 | 8 |  | 2011-2020 | 500 | 1995.4 (1989.4- 1996.7) |
| CRF55_01B | 2009-2013 | 4 |  | 2014-2020 | 49 | 2001.1 (1995.4- 2002.8) |

Note: * tMRCA denotes time to most recent common ancestor.

# HPD denotes highest posterior density.

@ The Guangxi historical reference sequences (n=61; sampled 1997-2013) were retrieved from Guangxi CDC institutional database to serve as tMRCA calibration.

+ The study samples were derived from 4,814-RNA sequence dataset.

Table S6 Bayesian Stochastic Search Variable Selection (BSSVS) results of main HIV subtypes/clusters transmission relationships between different age-gender groups in Qinzhou

|  |  | CRF01_AE cluster 1 | | CRF01_AE cluster 2 | | CRF07_BC | | CRF08_BC | |
| --- | --- | --- | --- | --- | --- | --- | --- | --- | --- |
| From | To | Mean counts | BF | Mean counts | BF | Mean counts | BF | Mean counts | BF |
| ≥70M | 18-24F | 0 | 0.8 | 0 | 0 | 0 | 0.5 | 0 | 1.4 |
| ≥70M | 25-34F | 0 | 0.6 | 0 | 0 | 0 | 0.4 | 0 | 1.0 |
| ≥70M | 35-49F | 0 | 0.8 | 0 | 0 | 0 | 0.4 | 0 | 0.4 |
| ≥70M | 50-69F | 0 | 0.8 | 0 | 0 | 3 | 12.3 | 0 | 1.1 |
| ≥70M | ≥70F | 0 | 3.0 | 0 | 0 | 2 | 13.9 | 1 | 7.9 |
| 50-69M | 18-24F | 0 | 0.2 | 11 | 670332.1 | 0 | 0.2 | 0 | 0.3 |
| 50-69M | 25-34F | 0 | 0.3 | 0 | 0 | 5 | 9.8 | 0 | 0.9 |
| 50-69M | 35-49F | 11 | 1620 | 15 | 670332.1 | 14 | 536630.2 | 0 | 0.1 |
| 50-69M | 50-69F | 72 | 573158.4 | 52 | 670332.1 | 59 | 536630.2 | 39 | 282614.9 |
| 50-69M | ≥70F | 6 | 255.0 | 8 | 670332.1 | 11 | 336.6 | 3 | 13.9 |
| 35-49M | 18-24F | 0 | 0.4 | 0 | 0 | 1 | 29.8 | 25 | 282614.9 |
| 35-49M | 25-34F | 19 | 114625.0 | 26 | 670332.1 | 7 | 48.4 | 22 | 282614.9 |
| 35-49M | 35-49F | 34 | 573158.4 | 0 | 0 | 2 | 24.8 | 42 | 282614.9 |
| 35-49M | 50-69F | 2 | 1.5 | 0 | 0 | 0 | 0.3 | 1 | 1.4 |
| 35-49M | ≥70F | 0 | 0.2 | 0 | 0 | 0 | 0.2 | 0 | 0.6 |
| 25-34M | 18-24F | 7 | 4434.9 | 4 | 670332.1 | 0 | 1.1 | 1 | 4.7 |
| 25-34M | 25-34F | 4 | 440.2 | 0 | 0 | 0 | 0.3 | 0 | 0.3 |
| 25-34M | 35-49F | 0 | 1.3 | 0 | 0 | 0 | 0.2 | 0 | 0.2 |
| 25-34M | 50-69F | 0 | 0.3 | 0 | 0 | 0 | 0.7 | 0 | 0.3 |
| 25-34M | ≥70F | 0 | 0.2 | 0 | 0 | 0 | 0.1 | 0 | 0.4 |
| 18-24M | 18-24F | 0 | 0.6 | 0 | 0 | 6 | 90.6 | 0 | 0.8 |
| 18-24M | 25-34F | 0 | 0.5 | 0 | 0 | 0 | 0.1 | 0 | 0.6 |

|  |  | CRF01_AE cluster 1 | | CRF01_AE cluster 2 | | CRF07_BC | | CRF08_BC | |
| --- | --- | --- | --- | --- | --- | --- | --- | --- | --- |
| From | To | Mean counts | BF | Mean counts | BF | Mean counts | BF | Mean counts | BF |
| 18-24M | 35- 49F | 0 | 0.5 | 0 | 0 | 0 | 0.4 | 0 | 0.6 |
| 18-24M | 50-69F | 0 | 0.5 | 0 | 0 | 0 | 0.6 | 0 | 0.5 |
| 18-24M | ≥70F | 0 | 0.5 | 0 | 0 | 0 | 0.1 | 0 | 0.5 |
| ≥70F | 18-24M | 0 | 1.1 | 0 | 0 | 0 | 1.2 | 0 | 1.1 |
| ≥70F | 25-34M | 0 | 1.3 | 0 | 0 | 0 | 1.2 | 0 | 1.0 |
| ≥70F | 35-49M | 0 | 1.4 | 0 | 0 | 0 | 1.4 | 0 | 1.5 |
| ≥70F | 50-69M | 0 | 1.1 | 0 | 0 | 0 | 1.3 | 0 | 1.3 |
| ≥70F | ≥70M | 0 | 1.3 | 0 | 0 | 0 | 1.3 | 0 | 0.8 |
| 50-69F | 18-24M | 0 | 1.9 | 0 | 0 | 0 | 0.5 | 0 | 0.7 |
| 50-69F | 25-34M | 0 | 1.1 | 0 | 0 | 0 | 0.7 | 0 | 0.8 |
| 50-69F | 35-49M | 0 | 0.8 | 0 | 0 | 0 | 1.1 | 0 | 1.4 |
| 50-69F | 50-69M | 1 | 2.4 | 0 | 0 | 0 | 2.2 | 0 | 1.0 |
| 50-69F | ≥70M | 0 | 0.6 | 3 | 670332.1 | 2 | 4.5 | 0 | 1.3 |
| 35-49F | 18-24M | 0 | 3.4 | 1 | 670332.1 | 0 | 0.6 | 0 | 0.9 |
| 35-49F | 25-34M | 0 | 1.7 | 0 | 0 | 0 | 0.7 | 0 | 0.6 |
| 35-49F | 35-49M | 0 | 0.8 | 0 | 0 | 0 | 1.7 | 0 | 1.2 |
| 35-49F | 50-69M | 0 | 1.0 | 0 | 0 | 0 | 0.7 | 0 | 1.1 |
| 35-49F | ≥70M | 0 | 0.6 | 0 | 0 | 0 | 0.5 | 0 | 0.5 |
| 25-34F | 18-24M | 0 | 0.7 | 1 | 670332.1 | 0 | 0.6 | 0 | 0.6 |
| 25-34F | 25-34M | 0 | 0.9 | 0 | 0 | 0 | 0.6 | 0 | 0.6 |
| 25-34F | 35-49M | 0 | 1.1 | 0 | 0 | 0 | 1.1 | 0 | 0.6 |
| 25-34F | 50-69M | 0 | 0.6 | 0 | 0 | 0 | 0.6 | 0 | 0.6 |
| 25-34F | ≥70M | 0 | 0.4 | 0 | 0 | 0 | 0.4 | 0 | 0.5 |

|  |  | CRF01_AE cluster 1 | | CRF01_AE cluster 2 | | CRF07_BC | | CRF08_BC | |
| --- | --- | --- | --- | --- | --- | --- | --- | --- | --- |
| From | To | Mean counts | BF | Mean counts | BF | Mean counts | BF | Mean counts | BF |
| 18-24F | 18-24M | 0 | 0.8 | 2 | 670332.1 | 0 | 0.5 | 0 | 4.1 |
| 18-24F | 25-34M | 0 | 0.5 | 0 | 0 | 0 | 0.5 | 0 | 2.1 |
| 18-24F | 35-49M | 0 | 0.4 | 0 | 0 | 0 | 0.5 | 0 | 0.4 |
| 18-24F | 50-69M | 0 | 0.4 | 0 | 0 | 0 | 0.5 | 0 | 0.3 |
| 18-24F | ≥70M | 0 | 0.3 | 0 | 0 | 0 | 0.4 | 0 | 0.4 |

Note: BF refers to Bayes Factor; All transmission events with decisive support (BF > 100) were associated with a posterior probability ≥ 0.9. F, female; M, Male;

This table presents the complete output of the BSSVS analysis; results with low BF values should be interpreted with caution as they indicate weaker statistical evidence for viral migration. All samples were derived from the 4814 RNA dataset.

Table S7 Global Moran’s I analysis of Mean Degree Centrality and Mean Genetic Cluster Growth across four distinct epidemiological phases

| Phase | Mean Degree Centrality | | | Mean Genetic Cluster Growth | | |
| --- | --- | --- | --- | --- | --- | --- |
|  | Global Moran I | z value | p value | Global Moran I | z value | p value |
| 1999-2009 | 0.298 | 4.316 | 0.000 | 0.314 | 4.235 | 0.000 |
| 2010-2015 | 0.184 | 2.460 | 0.007 | 0.223 | 2.950 | 0.002 |
| 2016-2018 | 0.208 | 2.753 | 0.003 | 0.205 | 2.699 | 0.003 |
| 2019-2021 | 0.115 | 1.584 | 0.057 | 0.373 | 4.765 | 0.000 |

Note: Samples were derived from the overall study samples (N=5,094)

Table S8 Questions and analysis of respondent driven sampling based behavioral survey

| Variable | Number | % |
| --- | --- | --- |
| Total | 84 | 100.0 |
| Q1. Age (years) |  | - |
| < 50 | 7 | 8.3 |
| 50-69 | 24 | 28.6 |
| ≥70 | 53 | 63.1 |
| Q2. Gender |  |  |
| Male | 82 | 97.6 |
| Q3. Marital status |  |  |
| Single | 15 | 17.9 |
| Married | 43 | 51.2 |
| Widow/divorced | 26 | 30.9 |
| Q4. Where are your social & recreational venues ?(Multiple-choice question) |  |  |
| Informal outdoor social gatherings (e.g., card playing at village entrances) | 63 | 75.0 |
| Community Park | 47 | 56.0 |
| Public little square | 46 | 54.8 |
| Traditional periodic markets | 40 | 47.6 |
| Street-side vending areas | 29 | 34.5 |
| Private residences of friends | 13 | 15.5 |
| Indoor recreational centers (Mahjong) | 11 | 13.1 |
| Indoor recreational centers (Chess parlors) | 7 | 8.3 |
| Q5. What is your primary source of income ? (Multiple-choice question) |  |  |
| Pension | 36 | 42.9 |
| Labor earnings | 43 | 51.2 |
| Children’s support | 41 | 48.8 |
| Minimum living guarantee subsidies | 8 | 9.5 |
| Q6. Have you ever engaged in casual or commercial heterosexual sex ? | 41 | 48.8 |
| Q7. Have you ever engaged in casual heterosexual sex ? |  |  |
| Yes | 20 | 23.8 |
| No | 64 | 76.2 |
| Q8. What is your number of casual heterosexual partners? [Median (IQR)] | 1.0 (1.0, 2.0) | - |
| Q9. Have you ever visited commercial sex venues ? |  |  |
| Yes | 38 | 45.2 |
| No | 46 | 54.8 |
| Q10. Have you ever engaged in commercial heterosexual sex ? |  |  |
| Yes | 36 | 42.9 |
| No | 48 | 57.1 |
| Q11. What about the locations of your commercial sex encounter ? (n=36) |  |  |
| Within township/street 2-km buffer of transportation arteries | 32 | 88.9 |
| Beyond township/street 2-km buffer of transportation arteries | 4 | 11.1 |
| Q12. What is the number of your commercial heterosexual partners? [Median (IQR)] | 2.0 (2.0, 4.0) | - |
| Q13. What is your frequency of visiting commercial or casual heterosexual partners ? (n=41) |  |  |
| ≥ Once weekly | 1 | 2.4 |
| > Once monthly to ≤ once weekly | 10 | 24.4 |
| > Once quarterly to ≤ once monthly | 7 | 17.1 |
| > Semi-annually to ≤ once quarterly | 7 | 17.1 |
| > Annually to ≤ semi-annually | 12 | 29.3 |
| Once only | 4 | 9.7 |
| Q14. How about your condom use with commercial or casual heterosexual partners ? (n=41) |  |  |
| Never | 13 | 31.7 |
| Occasionally | 13 | 31.7 |
| Frequently | 3 | 7.3 |
| Consistently | 12 | 29.3 |
| Q15. Are you aware of the typical price of commercial sex in your local venues? (CNY/transaction) (n=48) |  |  |
| < 50 | 18 | 37.5 |
| 50-100 | 17 | 35.4 |
| > 100 | 13 | 27.1 |
| Q16. What is the geographic origin of commercial sex workers you encountered (n=36) |  |  |
| Local (Within Qinzhou city) | 24 | 66.7 |
| Non-local (Within Guangxi, but outside Qinzhou) | 9 | 25.0 |
| Out-of-province (Outside Guangxi) | 3 | 8.3 |
| Q17. Were the commercial sex workers you encountered based permanently in a specific township’s venues? (n=36) |  |  |
| No. Non-fixed (Rotating among townships along transportation arteries, synchronized with market days) | 26 | 72.2 |
| Yes. Fixed (Permanently based within a single township). | 10 | 27.8 |

7,381 newly diagnosed HIV/AIDS cases

6,214 newly diagnosed HIV/AIDS cases with blood specimen

1. 714 cases were excluded due to failure of PCR^*^ amplification or Sequencing
2. 35 cases were excluded due to mixed bases ≥ 5%
3. 117 cases were excluded due to pol region < 1000bp or RT^#^ end < 238

5,348 **Qualified RNA or DNA sequences**

1. 38 cases excluded due to age < 18 years old
2. 19 cases excluded due to duplicated sampling
3. 197 cases excluded due to epidemiology information missing

5,094 cases with **qualified RNA or DNA sequences and complete epidemiological information**

**For HIV molecular network reconstruction and GEE^$^ analysis**

280 cases excluded for DNA sequences

4,814 cases with **qualified RNA sequences and complete epidemiological information**

Others (N=469)

CRF07BC (N=491)

CRF08BC (N=1,527)

CRF55_01B(N=49)

CRF01_AE cluster 1 (N=508)

CRF01_AE cluster 2 (N=1,770)

Subsampling

Subsampling

CRF01_AE cluster 2 (N=500)

CRF08BC (N=500)

**Bayesian Analysis**

Figure S1 Flowchart of study samples inclusion and exclusion

Note: * PCR denotes Polymerase Chain Reaction. # RT denotes Reverse Transcriptase. $ GEE refers to Generalized Estimating

Equations.


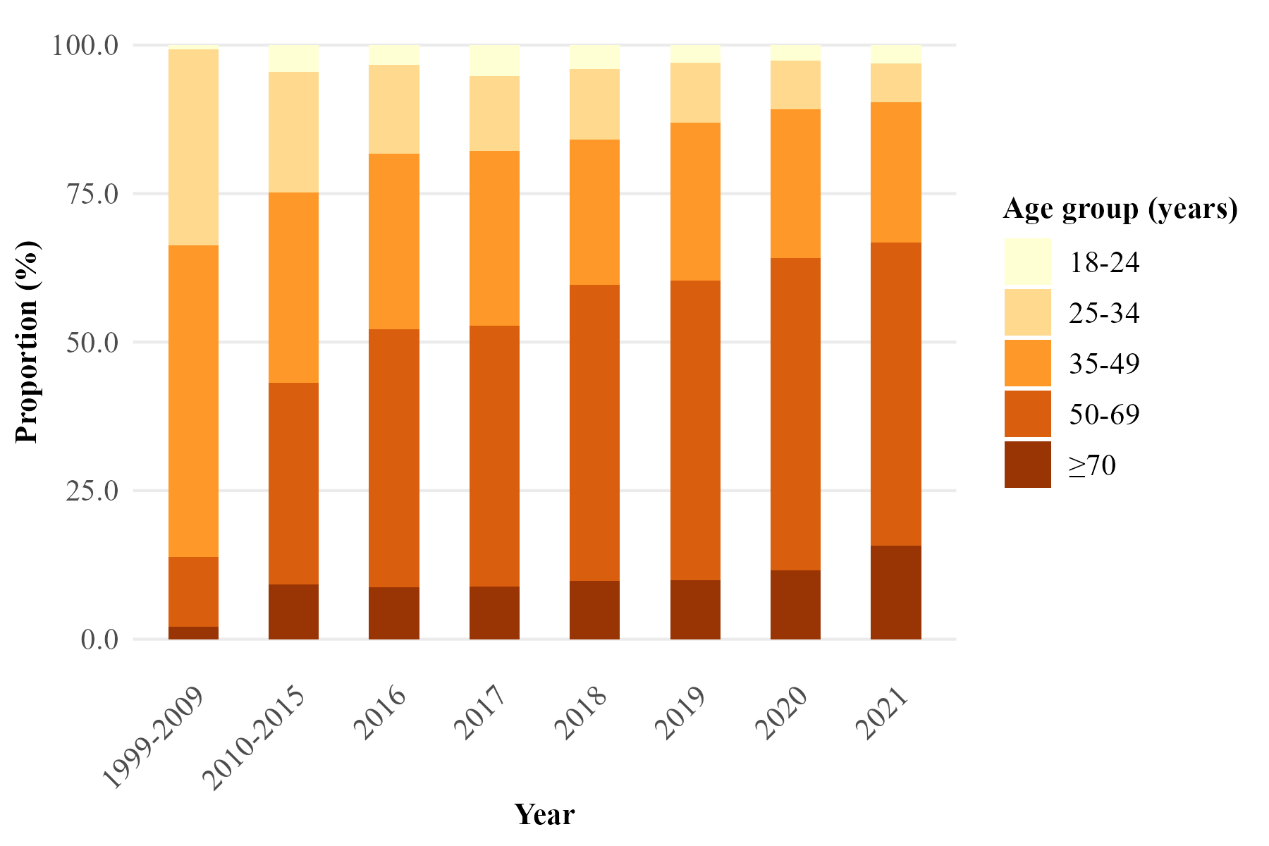

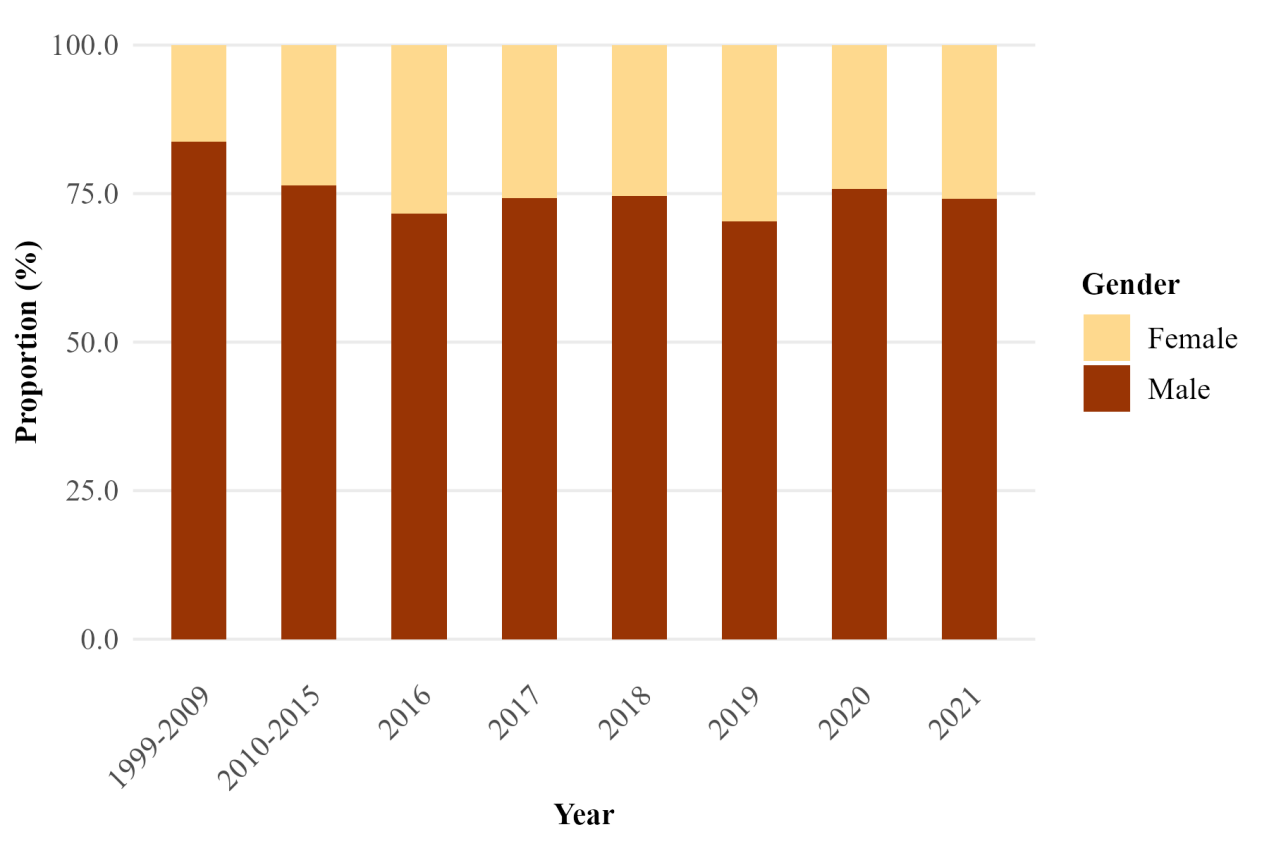


Figure S 2-1 Figure S 2-2


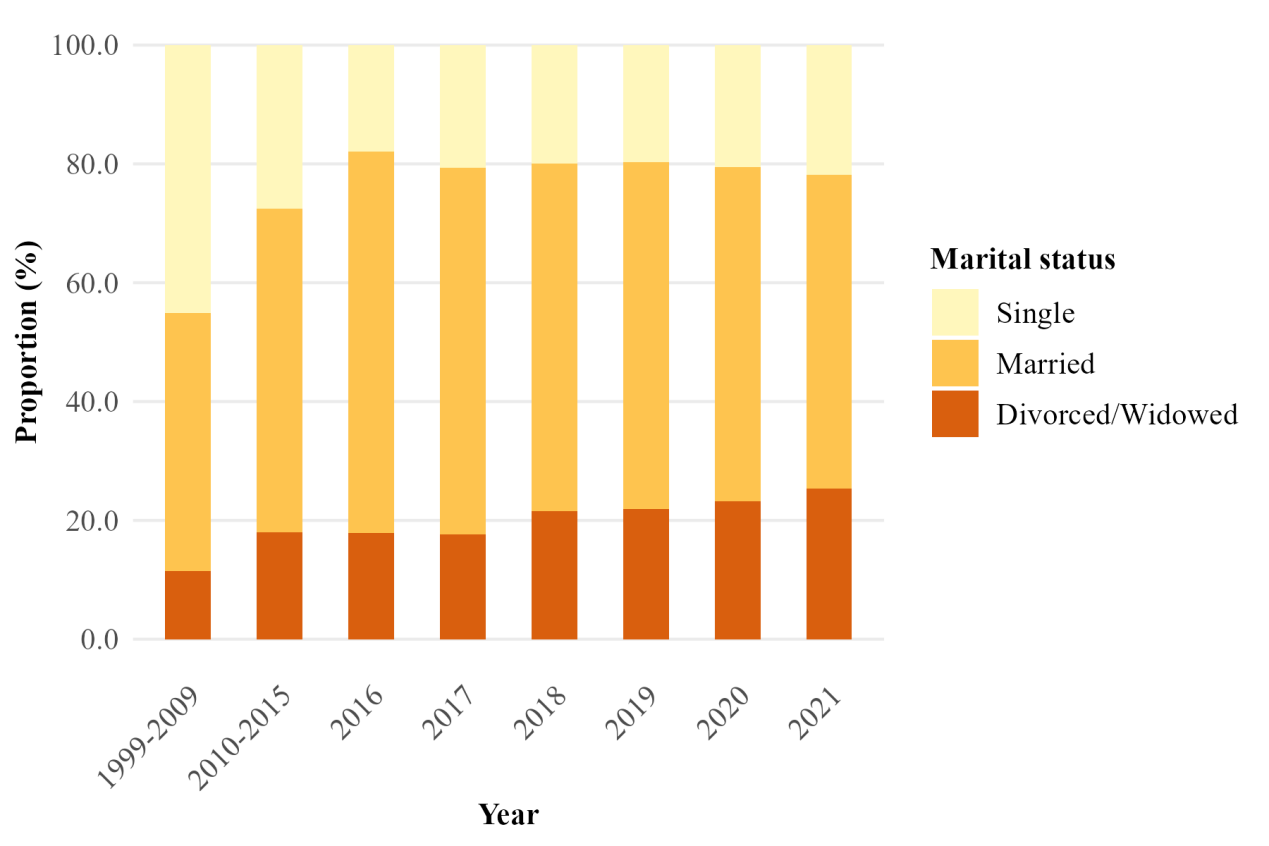

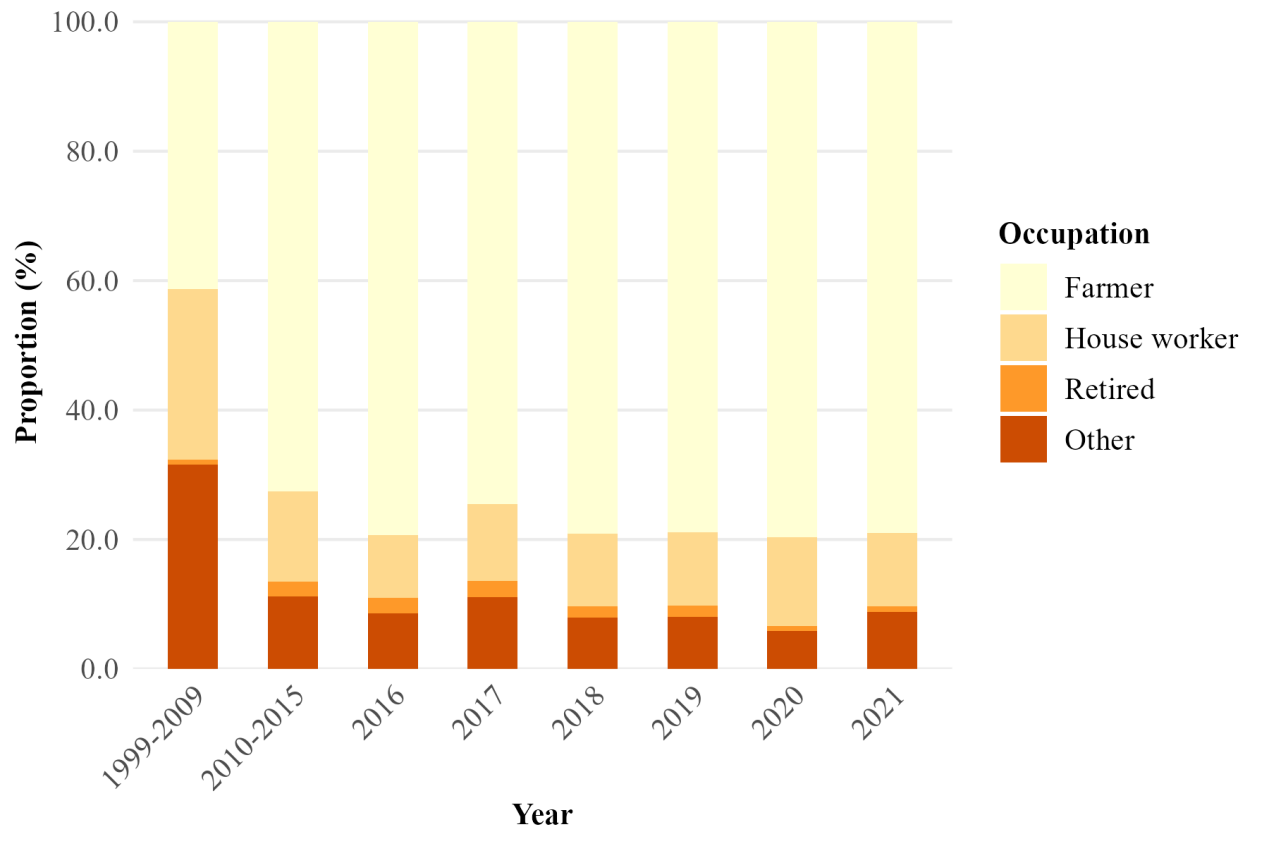


Figure S 2-3 Figure S 2-4


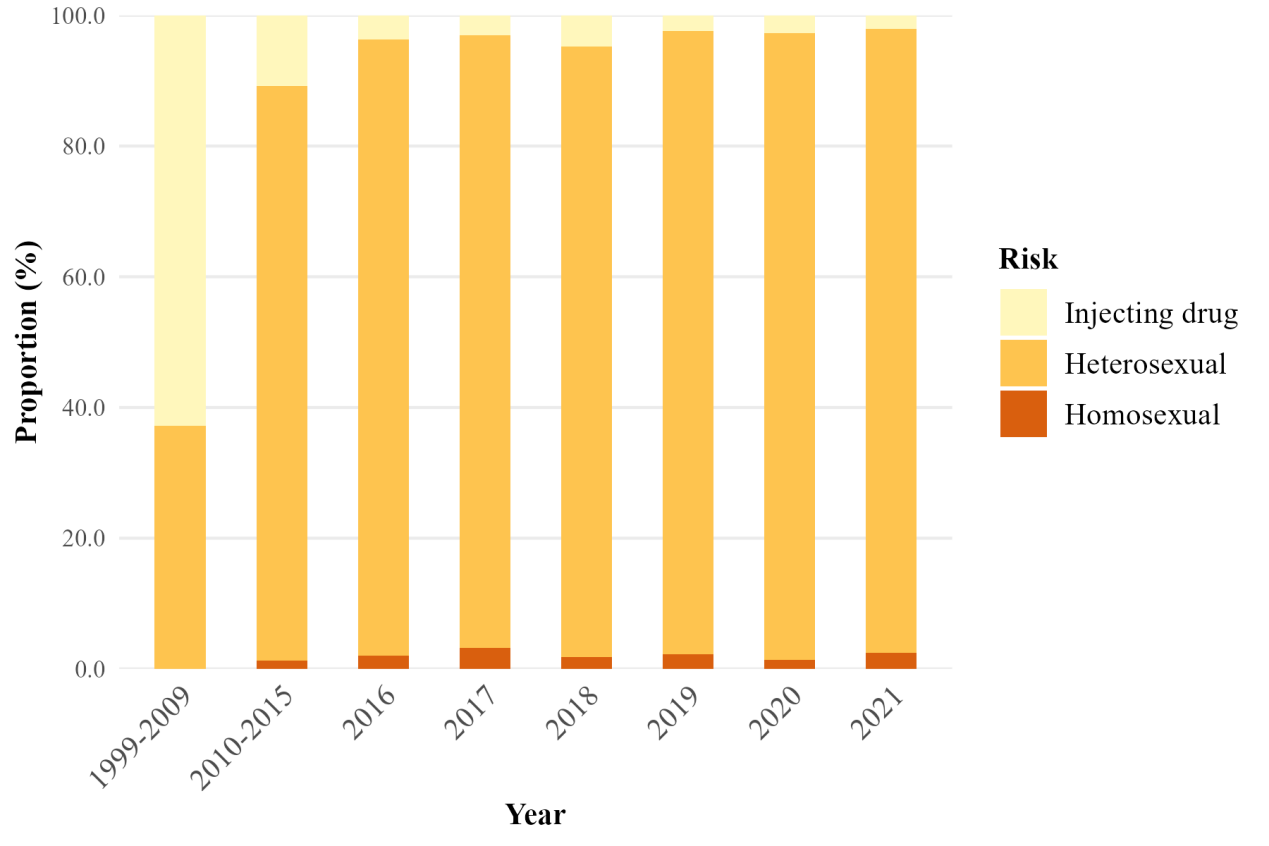

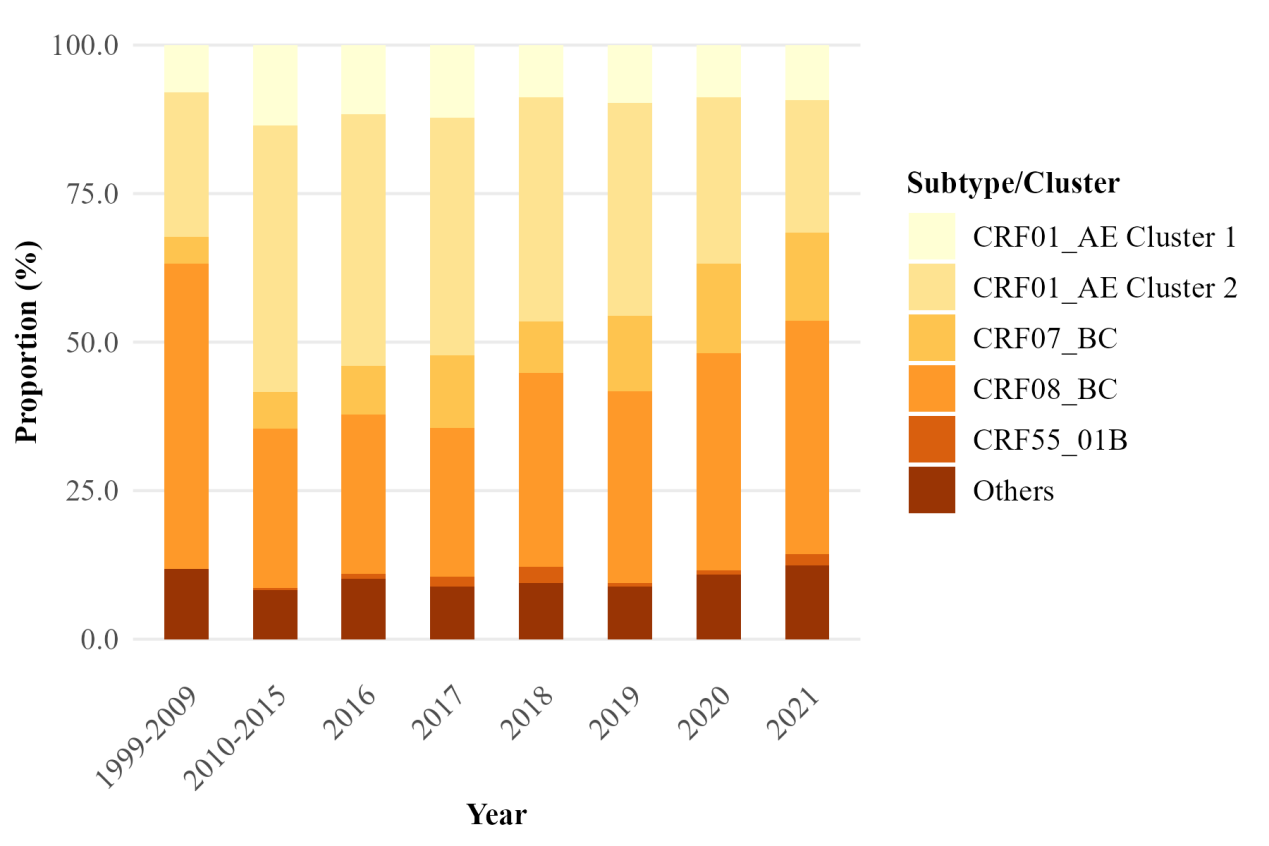


Figure S 2-5 Figure S 2-6

Figure S2 Temporal shifts in the proportional distribution of age, gender, marital status, occupation, risk and HIV-1 subtypes/clusters (1999–2021), visualized via 100% stacked column graphs. (N=5,094)


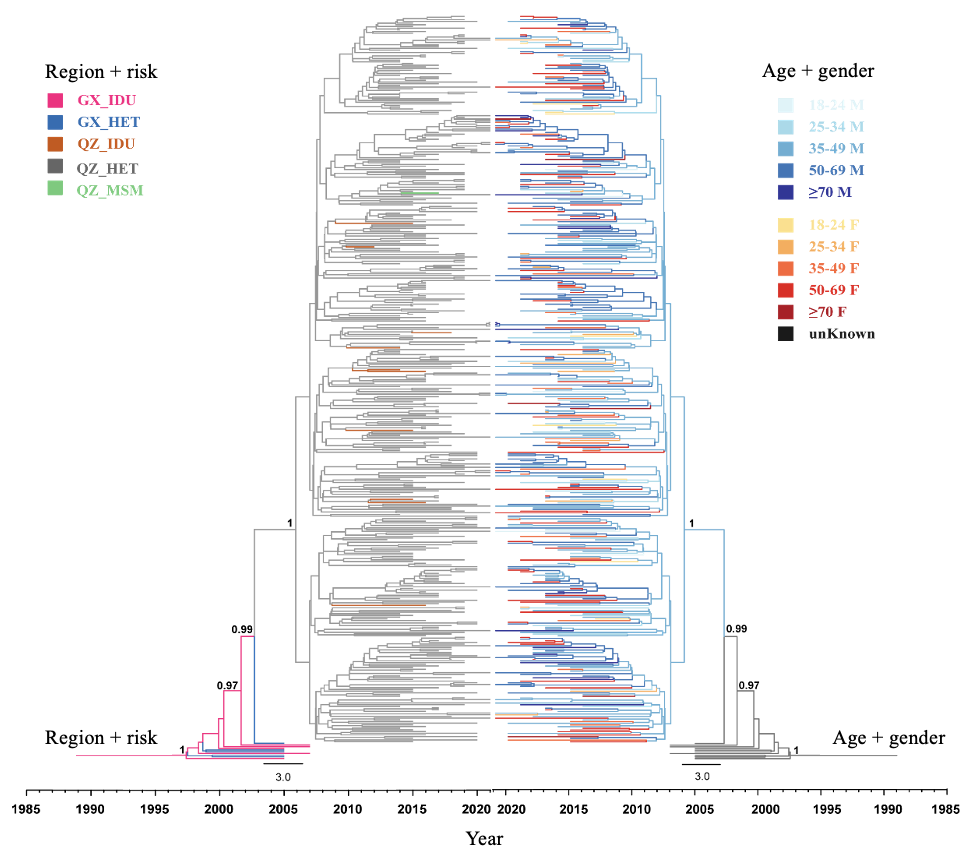


Figure S3-1 Maximum clade credibility tree of HIV-1 CRF01_AE cluster 1 transmission patterns. (A) Left panel: Phylogeographic reconstruction showing transmission sources by region and risk group (GX: Guangxi; QZ: Qinzhou). (B) Right panel: Demographic transmission patterns by age and gender (M: male; F: female).

Branches display posterior probabilities and nodes indicate divergence times (year-scale). Trees were time-scaled using Bayesian evolutionary analysis and visualized with FigTree v1.4.4. Scale bars denote evolutionary time in years. This dataset included 518 RNA sequences.


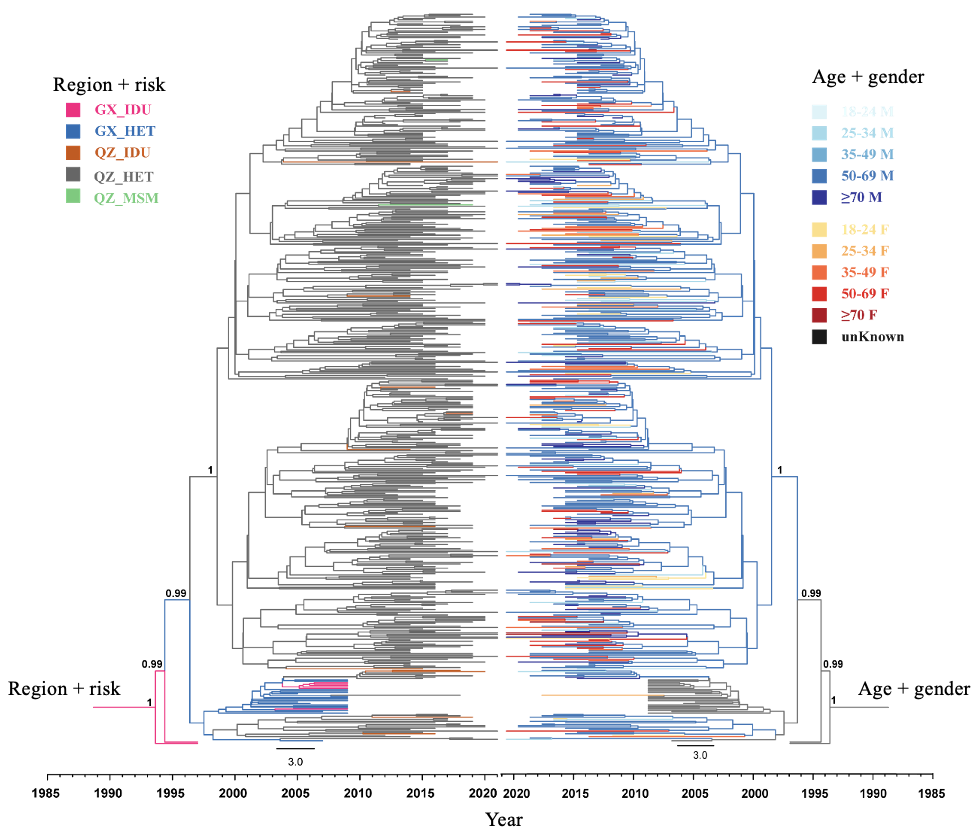


Figure S3-2 Maximum clade credibility tree of HIV-1 CRF01_AE cluster 2 transmission patterns. (A) Left panel: Transmission sources stratified by region and risk factors (GX: Guangxi; QZ: Qinzhou). (B) Right panel: Transmission patterns by age group and gender (M: male; F: female). Branches display posterior probabilities and nodes indicate divergence times (year-scale). Trees were reconstructed using Bayesian evolutionary analysis and visualized with FigTree (v1.4.4). Scale bars denote evolutionary time in years. This dataset included 527 RNA sequences.


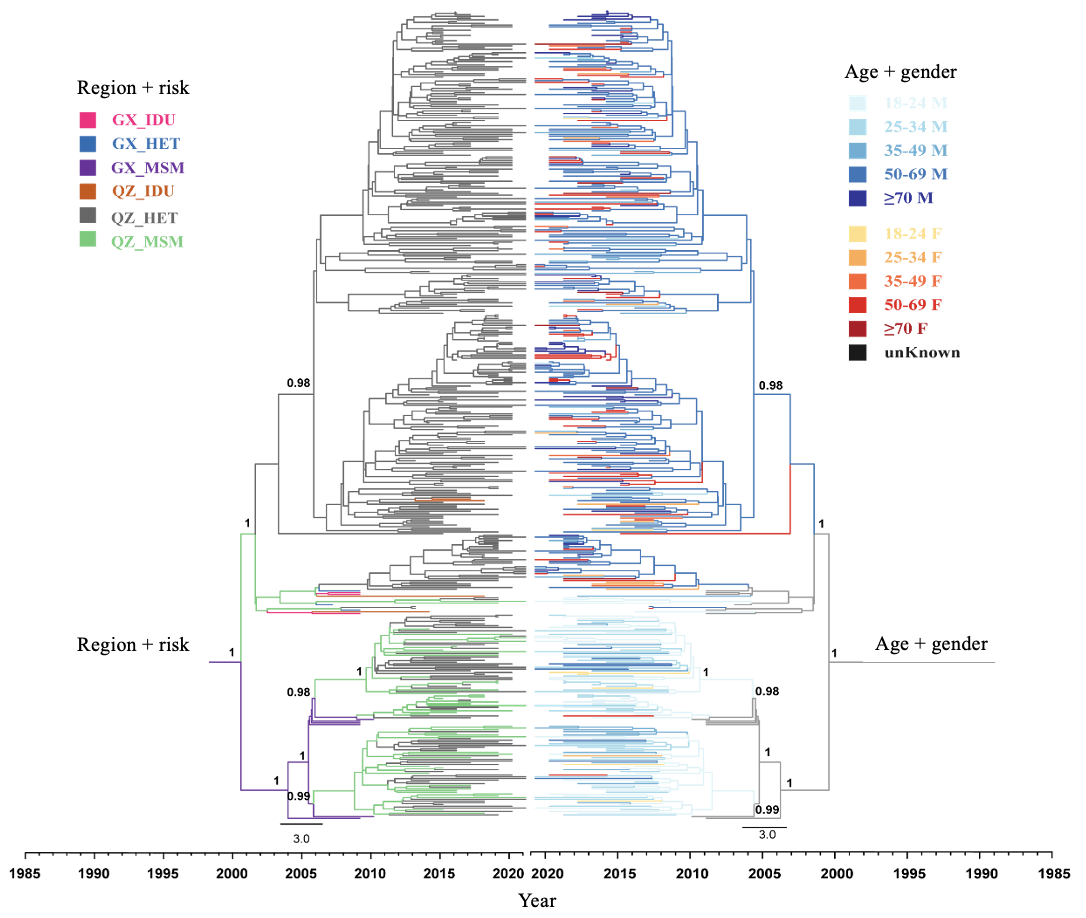


Figure S3-3 Maximum clade credibility tree of HIV-1 CRF07_BC transmission patterns. (A) Left panel: Transmission sources stratified by region and risk factors (GX: Guangxi; QZ: Qinzhou). (B) Right panel: Transmission patterns by age group and gender (M: male; F: female). Branches display posterior probabilities and nodes indicate divergence times (year-scale). Trees were reconstructed using Bayesian evolutionary analysis and visualized with FigTree (v1.4.4). Scale bars denote evolutionary time in years. This dataset included 503 RNA sequences.


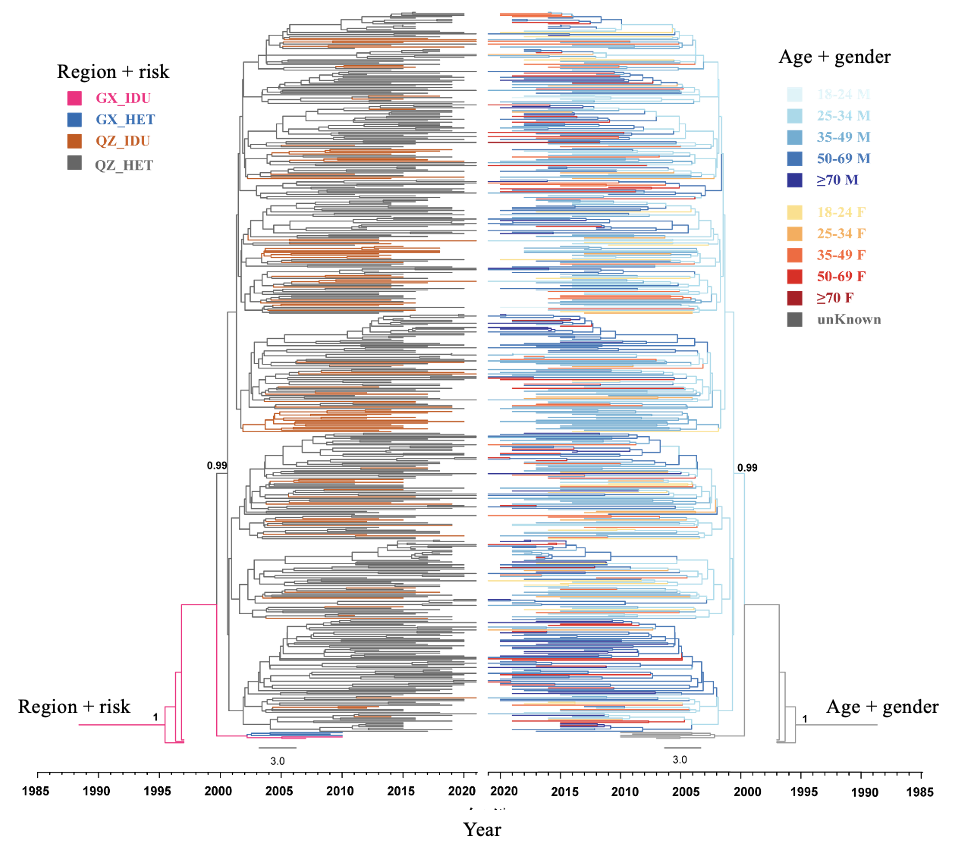


Figure S3-4 Maximum clade credibility tree of HIV-1 CRF08_BC transmission patterns. (A) Left panel: Transmission sources stratified by region and risk factors (GX: Guangxi; QZ: Qinzhou). (B) Right panel: Transmission patterns by age group and gender (M: male; F: female). Branches display posterior probabilities and nodes indicate divergence times (year-scale). Trees were reconstructed using Bayesian evolutionary analysis and visualized with FigTree (v1.4.4). Scale bars denote evolutionary time in years. This dataset included 508 RNA sequences.


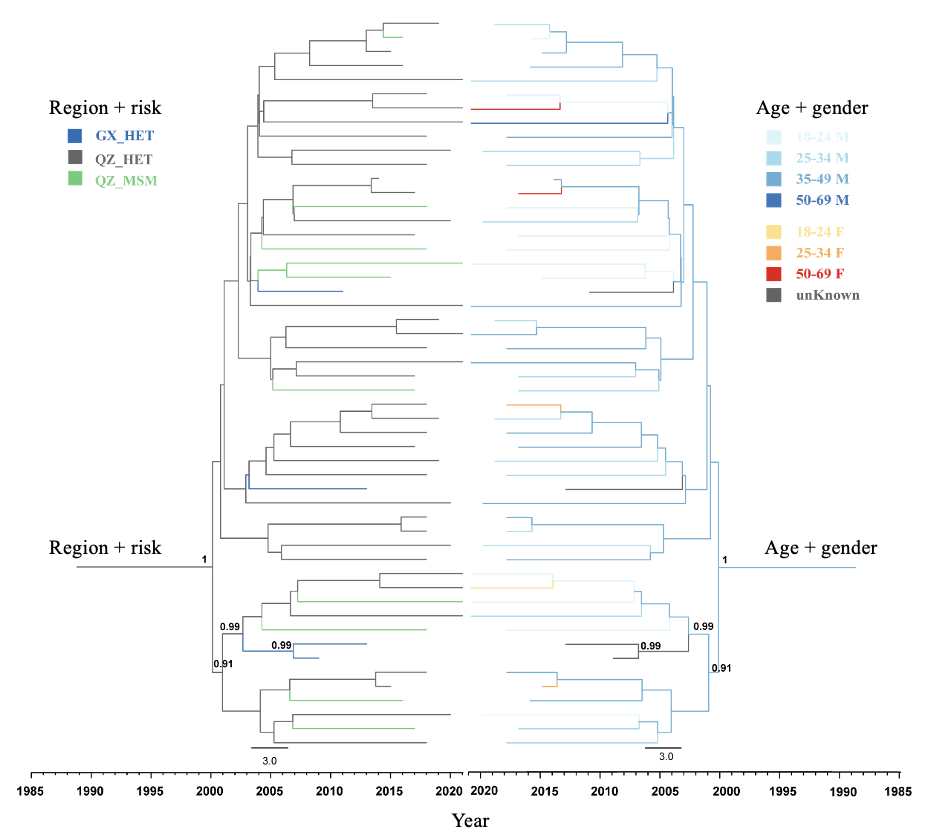


Figure S3-5 Maximum clade credibility tree of HIV-1 CRF55_01B transmission patterns. (A) Left panel: Transmission sources stratified by region and risk factors (GX: Guangxi; QZ: Qinzhou). (B) Right panel: Transmission patterns by age group and gender (M: male; F: female). Branches display posterior probabilities and nodes indicate divergence times (year-scale). Trees were reconstructed using Bayesian evolutionary analysis and visualized with FigTree (v1.4.4). Scale bars denote evolutionary time in years. This dataset included 53 RNA sequences.


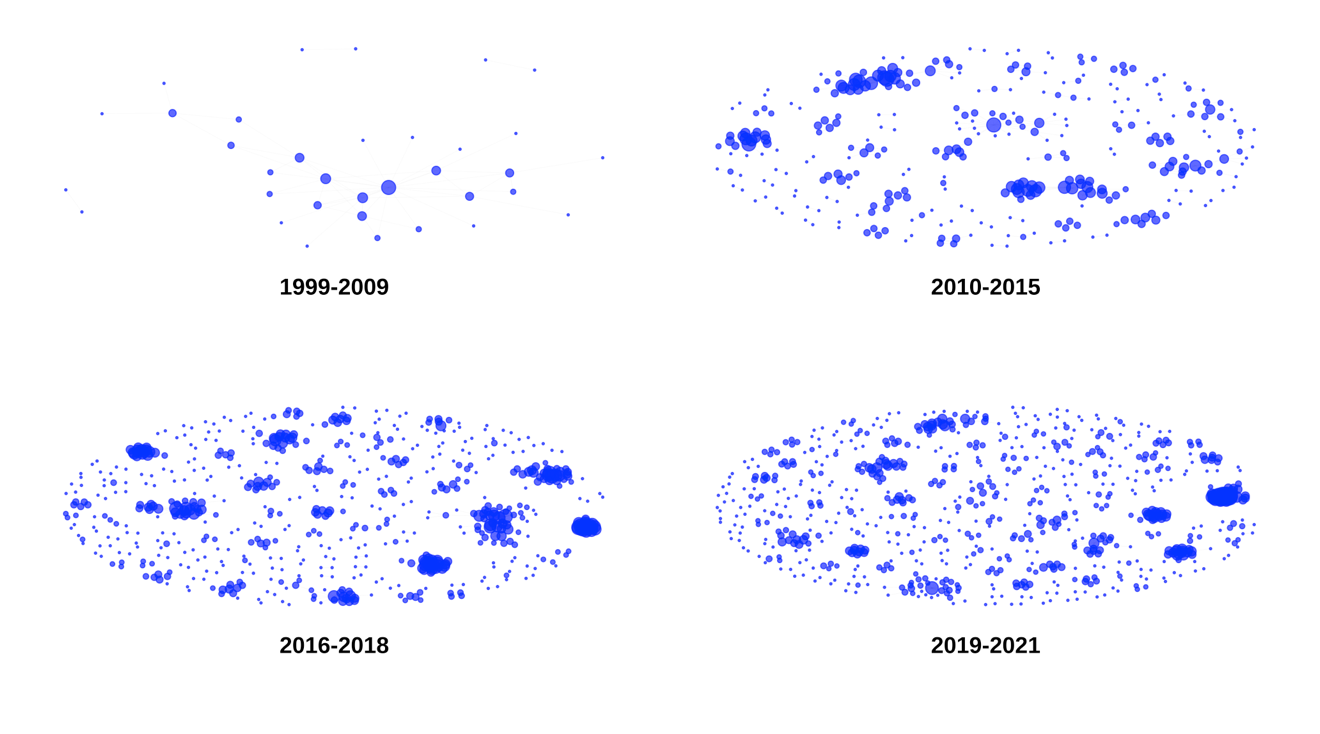


Figure S4 Phase-specific HIV-1 genetic networks (1999-2021). Nodes (blue dots) represent individual cases diagnosed in each phase, linked by a genetic distance threshold of 0.005 substitutions/site. The visualization highlights the evolving complexity of transmission clusters over time.


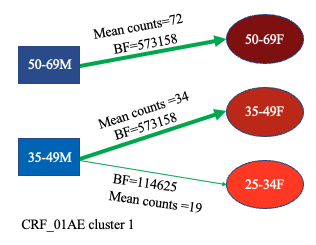

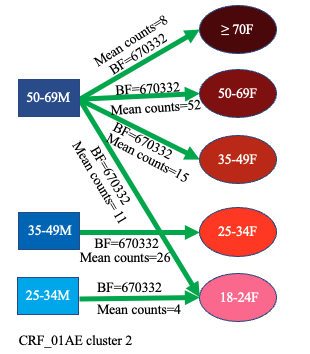


Figure S5-1 Figure S5-2


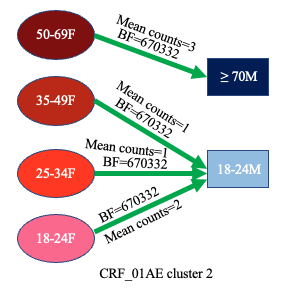

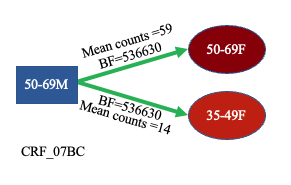


Figure S5-3 Figure S5-4


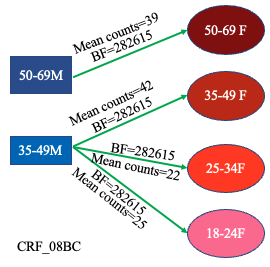


Figure S5 The transmission events between age-gender groups across HIV-1 subtypes. ≥70M denotes men at least 70 years old, 50-69M denotes men in 50-69 years old, 35-49M denotes men in 35-49 years old, 18-24F denotes women in 18-25 years old, 25-34F denotes women in 25-34 years old, 35-49F denotes women in 35-49 years old, 50-69F denotes women in 50-69 years old, ≥70F denotes women at least 70 years old. Only statistically robust transmission events with Bayes Factor (BF) ≥100,000 and posterior probability ≥ 0.9 were shown.

Figure S5-5
